# Augmenting neurogenesis rescues memory impairments in Alzheimer’s disease by restoring the memory-storing neurons

**DOI:** 10.1084/jem.20220391

**Published:** 2022-08-19

**Authors:** Rachana Mishra, Trongha Phan, Pavan Kumar, Zachery Morrissey, Muskan Gupta, Carolyn Hollands, Aashutosh Shetti, Kyra Lauren Lopez, Mark Maienschein-Cline, Hoonkyo Suh, Rene Hen, Orly Lazarov

**Affiliations:** 1 Department of Anatomy and Cell Biology, College of Medicine, The University of Illinois at Chicago, Chicago, IL; 2 Department of Psychiatry, College of Medicine, The University of Illinois at Chicago, Chicago, IL; 3 The Graduate Program in Neuroscience, The University of Illinois at Chicago, Chicago, IL; 4 Research Informatics Core, The University of Illinois at Chicago, Chicago, IL; 5 Department of Neurosciences, Cleveland Clinic, Cleveland, OH; 6 Department of Psychiatry, Irving Medical Center, Columbia University, New York, NY

## Abstract

Hippocampal neurogenesis is impaired in Alzheimer’s disease (AD) patients and familial Alzheimer’s disease (FAD) mouse models. However, it is unknown whether new neurons play a causative role in memory deficits. Here, we show that immature neurons were actively recruited into the engram following a hippocampus-dependent task. However, their recruitment is severely deficient in FAD. Recruited immature neurons exhibited compromised spine density and altered transcript profile. Targeted augmentation of neurogenesis in FAD mice restored the number of new neurons in the engram, the dendritic spine density, and the transcription signature of both immature and mature neurons, ultimately leading to the rescue of memory. Chemogenetic inactivation of immature neurons following enhanced neurogenesis in AD, reversed mouse performance, and diminished memory. Notably, AD-linked *App*, *ApoE*, and *Adam10* were of the top differentially expressed genes in the engram. Collectively, these observations suggest that defective neurogenesis contributes to memory failure in AD.

## Teaser

Deficits in hippocampal neurogenesis cause cognitive impairments in Alzheimer’s disease by compromising memory storage. Augmenting neurogenesis rescues memory by recruiting more new neurons with restored spine density and transcription profile. AD-linked genes regulate the memory-storing neurons.

## Graphical Abstract

**Figure:**
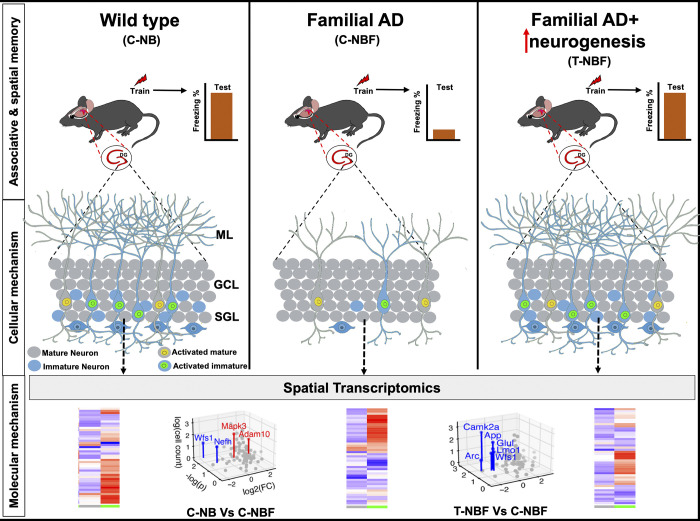

## Introduction

Alzheimer’s disease (AD) is characterized by progressive loss of memory and cognitive function. The mechanism underlying memory loss is largely unknown. Early memory impairments in AD affect episodic memory, spatial recognition, semantics, and visual orientation (Lazarov and Hollands, 2016). The dentate gyrus (DG) of the hippocampus plays a unique role as a neurogenic niche, where neural stem cells differentiate into new neurons that incorporate into its granular cell layer (Kempermann et al., 2015). In the adult brain, new dentate granule cells integrate into neural circuits and participate in hippocampal function (Anacker and Hen, 2017; Goncalves et al., 2016). The DG plays a role in memory acquisition, retrieval, and extinction of contextual fear conditioning (CFC; Bernier et al., 2017), with the latter two having distinct neural representations (Lacagnina et al., 2019). Immature neurons are implicated in hippocampal plasticity, contextual discrimination, and memory extinction (Akers et al., 2014; Gage, 2019; Sahay et al., 2011). Likewise, immature neurons were shown to be recruited into spatial memory networks (Kee et al., 2007). However, the role of immature neurons in memory formation is yet to be fully understood. Of particular interest is whether deficits in neurogenesis play a role in cognitive impairments, such as in AD. While previous work has shown that hippocampal neurogenesis is impaired in familial AD (FAD) mouse models and AD patients (Demars et al., 2010; Moreno-Jimenez et al., 2019; Tobin et al., 2019), a functional link between these impairments and memory deficits in AD is absent. Co-treatment of brain-derived neurotrophic factor (BDNF) and the modulation of Wnt3 signaling rescues the performance of FAD mice in the Y maze task (Choi et al., 2018). However, whether this effect is caused exclusively by immature neurons and what role they play in the memory circuit remains elusive. A previous study suggested that hippocampus-dependent memories are acquired successfully in a mouse model of FAD but cannot be retrieved and that optogenetic activation of memory engram cells led to memory retrieval (Roy et al., 2016). This study suggested that engram impairment is not due to deficits in new hippocampal neurons (Roy et al., 2016). Thus, we set to examine whether impaired engram formation is due to deficits in hippocampal neurogenesis in AD. Using virus-mediated engram labeling strategy, we show that the number of new neurons recruited into the memory engram in the FAD mice was significantly reduced compared to wild-type mice, their transcriptomic profile differed, and the density of dendritic spines of new neurons in the engram was impaired. Augmenting neurogenesis by *Nestin*-driven *CreER*^*T2*^ conditional *Bax* deletion in 5XFAD mice (Oakley et al., 2006; Sahay et al., 2011) rescued the number of new neurons recruited into the memory engram, restored the density of dendritic spines, and modulated the transcription profile of both immature and mature neurons, ultimately leading to restoration of contextual and spatial memory. Importantly, the chemogenetic inactivation of immature neurons in the DG of FAD mice with augmented neurogenesis caused memory deficits, suggesting that immature neurons are required for proper engram function, and their deficiency leads to engram malformation in AD, manifested by memory impairments. Notably, we show that the AD-linked genes, *ApoE*, *App*, and *Adam10*, exhibited the most significant change in engram cells between the groups. Taken together, these results suggest that augmenting neurogenesis may be of therapeutic value in AD and that AD-linked genes play a role in hippocampal memory formation.

## Results

### More new neurons in the DG of FAD mice result in better performance in hippocampus-dependent memory task

**Figure 1.**
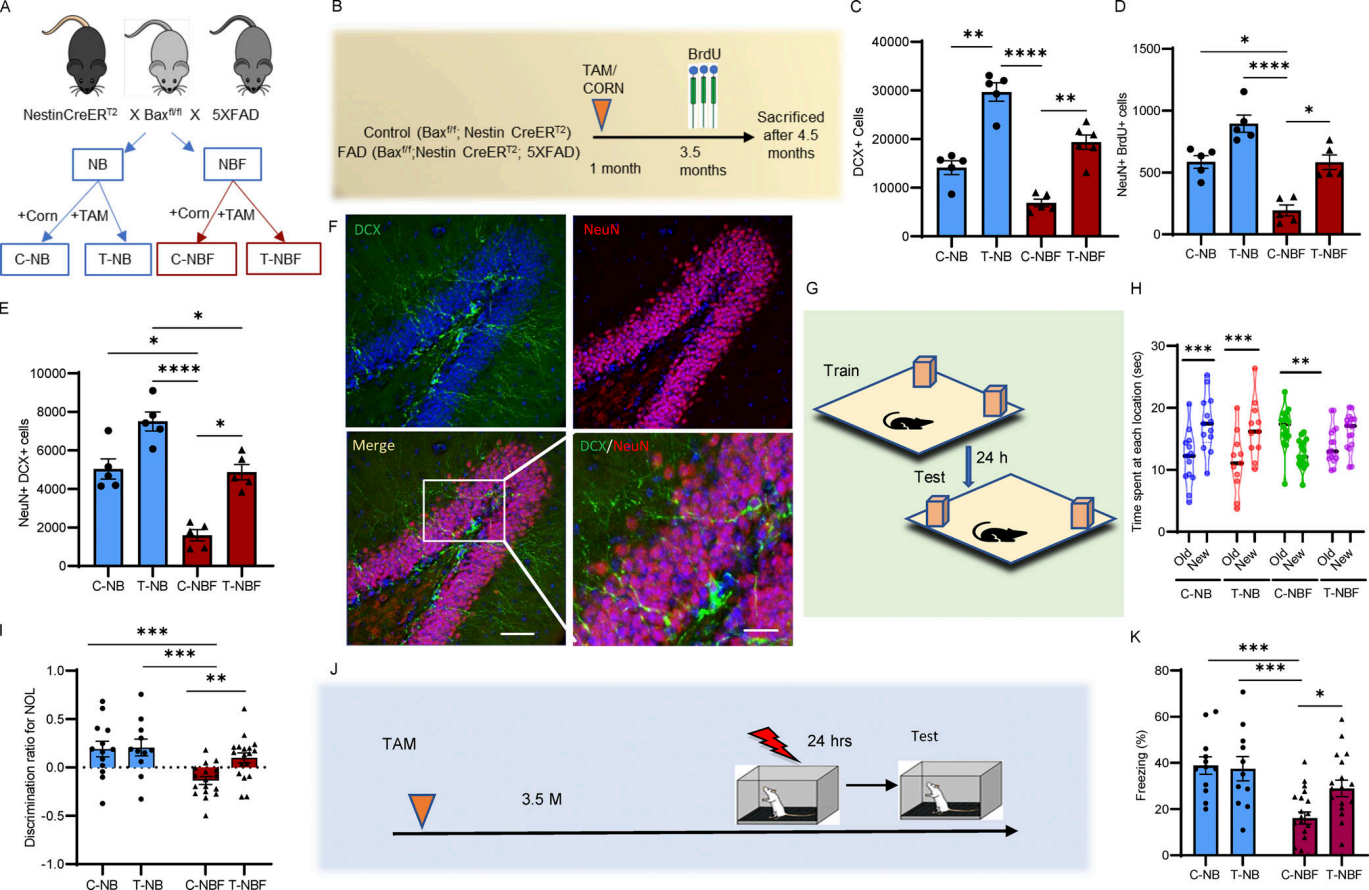
**Rescue of hippocampus-dependent memory following augmentation of neurogenesis in FAD mice. (A)** Breeding scheme. Abbreviations: corn oil (Corn, C)– or tamoxifen (TAM, T)-treated *NestinCreER*^*T2*^*; Bax*^*fl/fl*^ (NB) and *NestinCreER*^*T2*^*; Bax*^*fl/fl*^*;* 5XFAD (NBF). **(B)** Experimental paradigm. **(C)** The number of DCX-expressing neuroblasts and immature neurons in the DG of corn oil (C)– or tamoxifen (T)-treated *NestinCreER*^*T2*^*; Bax*^*fl/fl*^ (NB) and *NestinCreER*^*T2*^*; Bax*^*fl/fl*^*;* 5XFAD (NBF) quantified by unbiased stereology. *N* = 5 for C-NB, T-NB, and C-NBF; *N* = 6 for T-NBF; Kruskal–Wallis test with Dunn’s post hoc test (**P = 0.009, ****P = 0.0001). **(D)** The extent of survival of new neurons (BrdU^+^NeuN^+^) following treatment with either tamoxifen or corn in the DG of corn oil (C)– or tamoxifen (T)-treated *NestinCreER*^*T2*^*; Bax*^*fl/fl*^ (NB) and *NestinCreER*^*T2*^*; Bax*^*fl/fl*^*;* 5XFAD (NBF), as quantified by unbiased stereology. *N* = 5; *P < 0.05, ****P < 0.0001. **(E)** The number of new neurons (DCX^+^NeuN^+^) in the DG of corn oil (C)– or tamoxifen (T)-treated *NestinCreER*^*T2*^*; Bax*^*fl/fl*^ (NB) and *NestinCreER*^*T2*^*; Bax*^*fl/fl*^*;* 5XFAD (NBF), as quantified by unbiased stereology. *P < 0.05, ****P < 0.0001. **(F)** Confocal images of new neurons (DCX^+^NeuN^+^) in brain sections of tamoxifen (T)-treated *NestinCreER*^*T2*^*; Bax*^*fl/fl*^*;* 5XFAD (NBF). Scale bar = 50 μm; insert = 20 μm. **(G)** A scheme of the NOL test. **(H and I)** Performance of corn oil (C)– or tamoxifen (T)-treated *NestinCreER*^*T2*^*; Bax*^*fl/fl*^ (NB) and *NestinCreER*^*T2*^*; Bax*^*fl/fl*^*;* 5XFAD (NBF) in the NOL test. Results indicate the percentage of time spent at the novel location. One-way ANOVA with Fisher’s LSD post hoc test F(7, 104) = 6.032; P < 0.0001 and *P < 0.05, **P < 0.005 (H) and discrimination index. Kruskal–Wallis test with Dunn’s post hoc test; ***P = 0.009, **P = 0.005 (I). **(J)** Experimental design of the CFC test. **(K)** Percentage of freezing in the CFC test (***P < 0.001, *P = 0.012).

**Figure S1.**
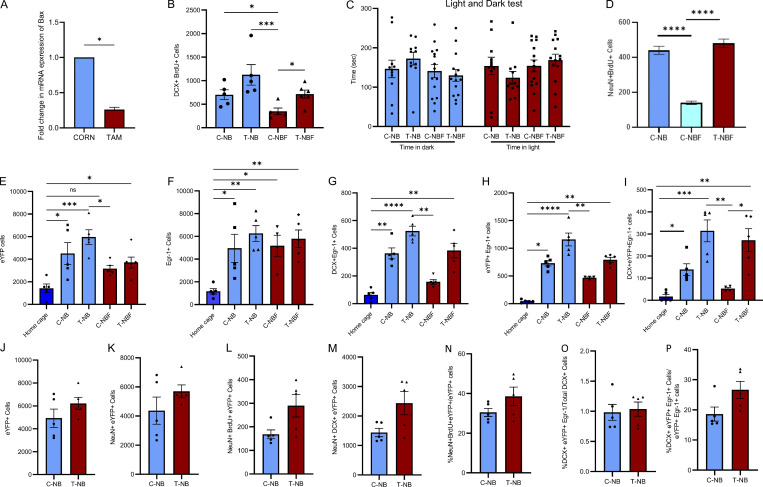
**Validation measures of experimental models. (A)** Bax expression level in neural progenitor cells cultured from the hippocampi of corn oil (C)– or tamoxifen (T)-treated *NestinCreER*^*T2*^*Bax*^*fl/fl*^ (NB) or *NestinCreER*^*T2*^*; Bax*^*fl/fl*^*;* 5XFAD (NBF) mice, as tested by PCR (Student’s *t* test, *P < 0.05). **(B)** The number of DCX^+^ and BrdU^+^ proliferating neuroblasts in the brains of corn oil (C)– or tamoxifen (T)-treated *NestinCreER*^*T2*^*Bax*^*fl/fl*^ (NB) or *NestinCreER*^*T2*^*; Bax*^*fl/fl*^*;* 5XFAD (NBF) mice 5 wk after BrdU injection. The number of proliferating neuroblasts was similar among most groups. A significant difference was observed between C-NB and C-NBF and T-NB and C-NBF (Kruskal–Wallis test with Dunn’s post hoc test; *P < 0.05, ***P = 0.0008). **(C)** 4.5-mo-old corn oil (C)– or tamoxifen (T)-treated *NestinCreER*^*T2*^*Bax*^*fl/fl*^ (NB) or *NestinCreER*^*T2*^*; Bax*^*fl/fl*^*;* 5XFAD (NBF) mice (*N* = 12–16) were subjected to the light–dark behavioral test. The time spent in the light or dark was comparable between the groups. Comparable anxiety levels were observed in wild type and FAD mice (two-way ANOVA with Fisher’s LSD post hoc test F(3, 94) = 0.009558, P = 0.9987). **(D)** Quantification of the number of surviving new neurons (BrdU^+^NeuN^+^) in mice that were not injected with viral engram cocktail (Kruskal–Wallis test with Dunn’s post hoc test; *P < 0.05). **(E–I)** Specificity of eYFP and Egr-1 expression in mouse brains following CFC. Quantification of the number of eYFP and Egr-1 expressing cells in brain sections of mice that were either maintained in standard housing (home cage) or underwent CFC. eYFP (E; Kruskal–Wallis test with Dunn’s post hoc test; *P < 0.05, ***P = 0.0001), Egr-1 (F: *P < 0.05, **P < 0.005), DCX^+^Egr-1^+^ (G: **P < 0.01, ****P < 0.0001), eYFP^+^Egr-1^+^ (H: *P = 0.0157, **P < 0.01, ****P < 0.0001), DCX^+^eYFP^+^Egr-1^+^ (I: *P < 0.05, **P < 0.01, ***P = 0.0002). **(J–P)** A comparison of the neurons in the engram in Corn oil- and tamoxifen-treated NestinCreER^T2^; Bax^fl/fl^ mice including total number of eYFP^+^ (J), NeuN^+^eYFP^+^ (K), NeuN^+^BrdU^+^eYFP^+^ (L), NeuN^+^DCX^+^eYFP^+^ (M), % NeuN^+^DCX^+^eYFP^+^/total eYFP^+^ (N), % DCX^+^eYFP^+^Egr-1^+^/total DCX^+^ (O), % DCX^+^eYFP^+^Egr-1^+^/total eYFP^+^Egr-1^+^ (P; P = ns, **P = 0.001).

To examine whether the augmentation of adult hippocampal neurogenesis rescues learning and memory deficits in FAD, we generated a mouse model of FAD with inducible neurogenesis. For this, we bred 5XFAD mice (Oakley et al., 2006) and wild-type counterparts with *NestinCreER*^*T2*^*; Bax*^*fl/fl*^ mice (Sahay et al., 2011; Fig. 1 A). Female *NestinCreER*^*T2*^*; Bax*^*fl/fl*^ (NB) or *NestinCreER*^*T2*^*; Bax*^*fl/fl*^*; *5XFAD (NBF) mice were treated with either tamoxifen (T-NB or T-NBF) or corn oil at 1 mo of age (C-NB or C-NBF, respectively; Fig. 1 B). Recombination-induced deletion of *Bax* was verified by qPCR revealing 75% reduction in *Bax* (Fig. S1 A). In support of the previous report, *Bax* deletion enhanced the survival of neural progenitor cells (NPCs) and led to increased neurogenesis (Sahay et al., 2011). The number of proliferating neuroblasts was trending but not significantly increased following recombination (Fig. S1 B), possibly because the deletion of *Bax* enhances the survival of these cells, rather than their rate of proliferation, and thus would be manifested mainly by an increase in the number of immature and new neurons. Notably, a significantly lower number of proliferating neuroblasts was observed in the C-NBF mice in comparison to the C-NB mice, supporting the notion that neurogenesis decreases in AD (Fig. S1 B; *P < 0.05, ***P = 0.0008). In support of that, T-NB had significantly higher number of immature neurons (doublecortin [DCX]^+^; Fig. 1 C; *N* = 5 for C-NB, T-NB, and C-NBF; *N* = 6 for T-NBF; **P = 0.009, ****P = 0.0001), greater survival of new neurons (BrdU^+^NeuN^+^; Fig. 1 D; *N* = 5; *P = 0.04, ****P < 0.0001), as well as more immature neurons (DCX^+^NeuN^+^; Fig. 1, E and F; *P < 0.05, ****P < 0.0001) compared to age- and gender-matched C-NB mice. C-NBF had significantly fewer immature neurons and reduced survival and number of new neurons compared to C-NB (Fig. 1, C–F), validating that this model recapitulates the impairments in hippocampal neurogenesis in 5XFAD (Moon et al., 2014; Zaletel et al., 2018). Importantly, T-NBF mice had significantly more immature and new neurons compared to C-NBF (Fig. 1, C–F). To examine whether increased neurogenesis rescues memory impairments in FAD mice and whether immature neurons actively participate in memory malformation in the disease, the four groups of mice were subjected to a novel object location (NOL) paradigm, representing spatial recognition memory. C-NB and T-NB performed similarly well with 60% of their time spent on exploring the object in the novel location (Fig. 1, G and H; one-way ANOVA with Fisher’s least significant difference (LSD) post hoc test F(7, 104) = 6.032; P < 0.0001 and *P < 0.05, **P < 0.005). Likewise, their discrimination index was similar (Fig. 1 I; ***P = 0.009, **P = 0.005). This may suggest either a ceiling effect or that the behavior paradigm has limited sensitivity in detecting discrete improvement of memory performance. Notably, C-NBF mice failed the test, preferably exploring the old location, manifested by a negative discrimination index (Fig. 1, H and I). Importantly, T-NBF mice showed significantly improved performance with increased preference for the novel location and a greater discrimination index similar to the NB mice (Fig. 1, H and I). To further characterize the effect of enhanced neurogenesis on memory in AD, another cohort of C-NB, T-NB, C-NBF, and T-NBF mice were subjected to a different behavioral paradigm examining associative memory using the hippocampus-dependent CFC test (Fig. 1 J; Kim and Fanselow, 1992; Remaud et al., 2014). Comparable to their performance in the NOL test, C-NB and T-NB mice were able to associate the context with the shock similarly well with an average freezing of 40% on the test day (Fig. 1 K; ***P < 0.001, *P = 0.01). C-NBF mice performed poorly on this test, with a 20% average freezing on the testing day (Fig. 1 K). Importantly, T-NBF mice associated the context with the shock significantly better with 30% average freezing (Fig. 1 K). To further corroborate the above finding, we characterized mouse anxiety levels by subjecting them to a light–dark test. The four groups of mice showed comparable levels of anxiety (Fig. S1 C). Taken together, these results strongly suggest that augmenting hippocampal neurogenesis in FAD mice significantly improves spatial recognition and contextual memory.

### New neurons play a role in memory acquisition in FAD

**Figure 2.**
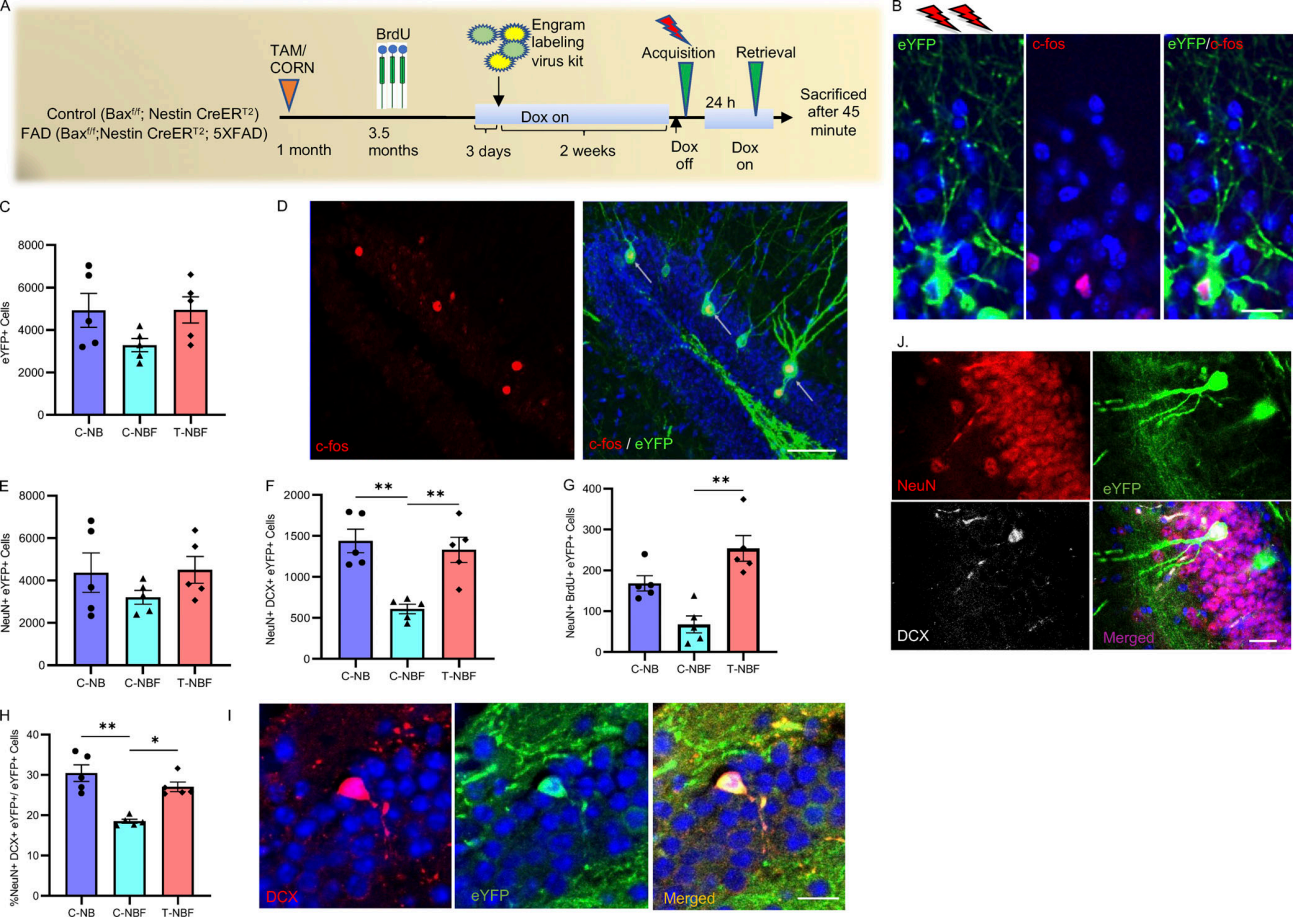
**Augmenting neurogenesis rescues the recruitment of immature neurons into the memory circuit. (A)** Experimental design aiming to identify immature neurons incorporating in the contextual memory circuit in the DG of corn oil (C)–treated *NestinCreER*^*T2*^*; Bax*^*fl/fl*^ (NB) and corn oil (C)– or tamoxifen (T)-treated *NestinCreER*^*T2*^*; Bax*^*fl/fl*^*;* 5XFAD (NBF) mice. **(B)** Confocal images demonstrating the recruitment of a granule neuron into the memory circuit during memory acquisition (eYFP) and retrieval (c-fos). eYFP expression in granule neurons infected with the viral engram kit AAV9-cFos-tTA and AAV9-TRE-ChR2-eYFP in the DG of *NestinCreER*^*T2*^*; Bax*^*fl/fl*^ mice was upregulated following CFC training (shock). c-fos was upregulated following the test. Brain sections were immunostained with antibodies raised against c-fos and show co-localization of c-fos and eYFP. Scale bar = 20 μm. **(C and E–G)** Quantification of cells incorporated in memory circuit during acquisition. Total number of activated cells (C; eYFP^+^; Kruskal–Wallis test with Dunn’s post hoc test, P = ns); total number of activated neurons (E; NeuN^+^eYFP^+^; P = ns); number of new neurons (F; NeuN^+^DCX^+^eYFP^+^; **P < 0.01). The number of new neurons born 6 wk prior to CFC (G; NeuN^+^BrdU^+^eYFP^+^; **P = 0.0011) in the DG of corn oil (C)–treated *NestinCreER*^*T2*^*;* *Bax*^*fl/fl*^ (NB) and corn oil (C)– or tamoxifen (T)-treated *NestinCreER*^*T2*^*;* *Bax*^*fl/fl*^*;* 5XFAD (NBF). **(D)** Co-localization of enhanced eYFP and endogenous c-fos in granule neurons in the DG of tamoxifen (T)-treated *NestinCreER*^*T2*^*; Bax*^*fl/fl*^*;* 5XFAD (NBF) mouse. Scale bar = 75 μm. **(H)** Percentage of new neurons recruited into the engram to the total eYFP^+^ cells (*P = 0.0284 and **P = 0.0019). **(I and J)** Co-localization of eYFP and DCX^+^ (I), and eYFP^+^, DCX^+^, and NeuN^+^ (J) in immature neurons in the DG of tamoxifen (T)-treated *NestinCreER*^*T2*^*; Bax*^*fl/fl*^*;* 5XFAD (NBF) mouse. Scale bar = 20 μm.

To address whether immature neurons rescue memory in FAD by participating in memory formation, we sought to track neurons that were activated following memory acquisition. For this purpose, we used a Tet-off–based viral engram labeling kit composed of a cocktail of AAV9-cFos-tTA and AAV9-TRE-ChR2-eYFP (a gift from Dr. Susumu Tonegawa, Massachusetts Institute of Technology, Cambridge, MA; Roy et al., 2016). Mice were put on a doxycycline diet starting 1 wk before the stereotactic injection of the cocktail into the DG and taken off 18 h before foot shock administration to allow the expression of enhanced yellow fluorescent protein (eYFP) in activated neurons during memory acquisition (Fig. 2, A and B). Co-immunostaining with anti–c-fos antibodies validated that eYFP^+^ neurons expressed c-fos, suggesting that these neurons were recruited into the neuronal ensemble during memory acquisition and reactivated during memory retrieval (Fig. 2, B and D). A previous study reports that certain serotypes of AAV induce massive death of BrdU-labeled cells within 18 h of injection and no evidence of recovery of adult neurogenesis at 3 mo after injection (Johnston et al., 2021). To examine whether the viral engram labeling kit affected the extent of neurogenesis in the current study, we compared the number of surviving immature neurons (NeuN^+^BrdU^+^) in mice pre-injected with the viral vectors (Fig. 1 D) and in mice that were not injected (Fig. S1 D). We observed comparable numbers in both groups of mice. To further validate the specificity of our labeling approach, the numbers of eYFP^+^, Egr-1^+^, eYFP^+^Egr-1^+^, DCX^+^ eYFP^+^, DCX^+^Egr-1^+^, and DCX^+^eYFP^+^Egr-1^+^ cells were assessed in mice that were kept in their home cage. We observed a significantly lower number of activated cells in the DG of these mice compared to mice subjected to CFC, suggesting that the observed recruitment of these cells into the memory circuit is highly specific to the CFC (Fig. S1, E–I). To examine whether new neurons participate in memory acquisition and whether increased neurogenesis in FAD mice results in greater recruitment of new neurons into the memory circuit, we first quantified the total number of eYFP^+^ cells in the brains of the four groups of mice. In agreement with the behavior, we observed a similar number of cells recruited into the engram in C-NB and T-NB (Fig. S1, J–P). Thus, further quantification was focused on the engram of behaviorally impaired FAD mice and its state following augmentation of neurogenesis (T-NBF). The total number of cells recruited into the engram following CFC training (i.e., eYFP^+^ cells) was similar in the C-NB, C-NBF, and T-NBF mice (Fig. 2 C; P = 0.1039). To examine the total number of eYFP^+^ neurons, we co-stained brain sections with anti-NeuN antibodies. We observed that the numbers of NeuN^+^eYFP^+^ were similar to total eYFP^+^ cells in all groups, suggesting that cells recruited following memory acquisition in the DG were neurons. The number of total NeuN^+^ eYFP^+^ was comparable between the experimental groups (Fig. 2 E). Interestingly, marked differences were observed between the groups in the number of new neurons recruited into the engram. Specifically, the number of new neurons recruited into the circuit (NeuN^+^DCX^+^eYFP^+^) was significantly compromised in the C-NBF mice compared to the C-NB mice (Fig. 2, F, I, and J; **P = 0.0072). Notably, the number of activated new neurons NeuN^+^DCX^+^eYFP^+^ was significantly increased in T-NBF mice (Fig. 2 F; **P = 0.0089). Similar results were observed in analyzing the rate of survival of new neurons (NeuN^+^BrdU^+^eYFP^+^) recruited into the memory circuit in the different groups (Fig. 2 G; **P = 0.0011). Notably, the percentage of activated new neurons to the total activated neurons revealed a significantly reduced percentage in the C-NBF group compared to the C-NB and restoration in the T-NBF group (%(NeuN^+^ DCX^+^ eYFP^+^)/total eYFP^+^ cells; Fig. 2 H; **P = 0.0019, *P = 0.0284). Taken together, these results suggest that fewer immature neurons were recruited into the engram during memory acquisition in C-NBF and that augmenting neurogenesis led to the recruitment of more immature neurons, comparable to their numbers in C-NB mice. Furthermore, these results suggest that a reduced number of immature neurons recruited into the engram correlated with impaired behavior in C-NBF, whereas an increased number correlated with intact performance in T-NBF mice.

### Fewer neurons recruited during memory acquisition get reactivated at retrieval in FAD

**Figure 3.**
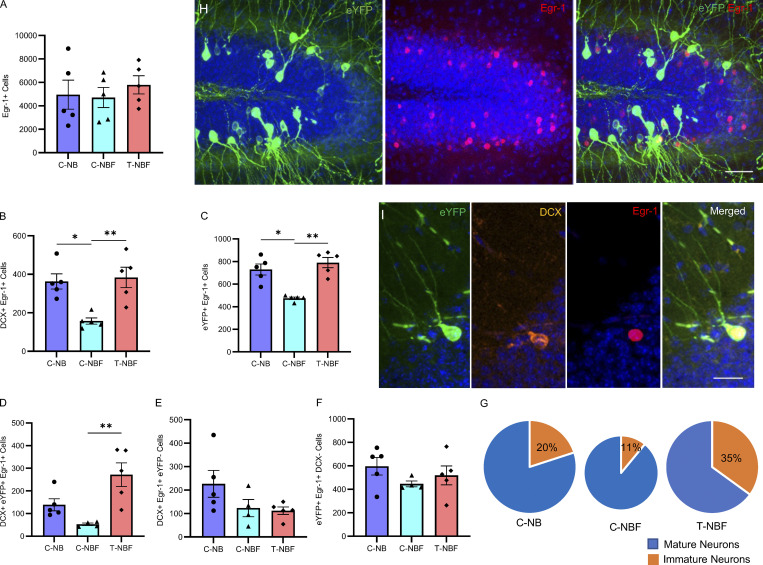
**Enhanced number of immature neurons reactivated in the retrieval of contextual memory in FAD mice following augmented neurogenesis. (A–G)** Quantification of the number of cells recruited into the contextual memory circuit in corn oil (C)– or tamoxifen (T)-treated *NestinCreER*^*T2*^*; Bax*^*fl/fl*^*;* 5XFAD (NBF) compared to corn oil–treated *NestinCreER*^*T2*^*; Bax*^*fl/fl*^ (C-NB) mice following memory retrieval on test day of CFC using unbiased stereology. **(A)** Total number of cells (Egr-1^+^). **(B)** New neurons recruited during memory retrieval (DCX^+^Egr-1^+^; Kruskal–Wallis test with Dunn’s post hoc test; *P =0.0109, **P = 0.0058). **(C)** Total number of cells incorporated in both memory acquisition and reactivated in memory retrieval (eYFP^+^Egr-1^+^; *P = 0.0162, **P = 0.0037). **(D)** New neurons incorporated in both memory acquisition and reactivated in memory retrieval (DCX^+^eYFP^+^Egr-1^+^; **P = 0.0015). **(E)** New neurons incorporated in memory retrieval but not during acquisition (DCX^+^Egr-1^+^eYFP^−^). **(F)** Mature granule neurons incorporated in both memory acquisition and reactivated in memory retrieval (DCX^−^Egr-1^+^eYFP^+^; P = ns). **(G)** The portion of new and mature granule neurons in the contextual memory engram in the three experimental groups. Pie graphs were scaled based on the total size of the engram in each experimental group. **(H and I)** Confocal images of eYFP^+^ and Egr-1^+^ cells in the DG of mice injected with engram kit cocktail (H), scale bar = 50 μm; and a representative new neuron (DCX^+^) co-expressing eYFP^+^ and Egr-1^+^ (I), scale bar = 20 μm.

**Figure S2.**
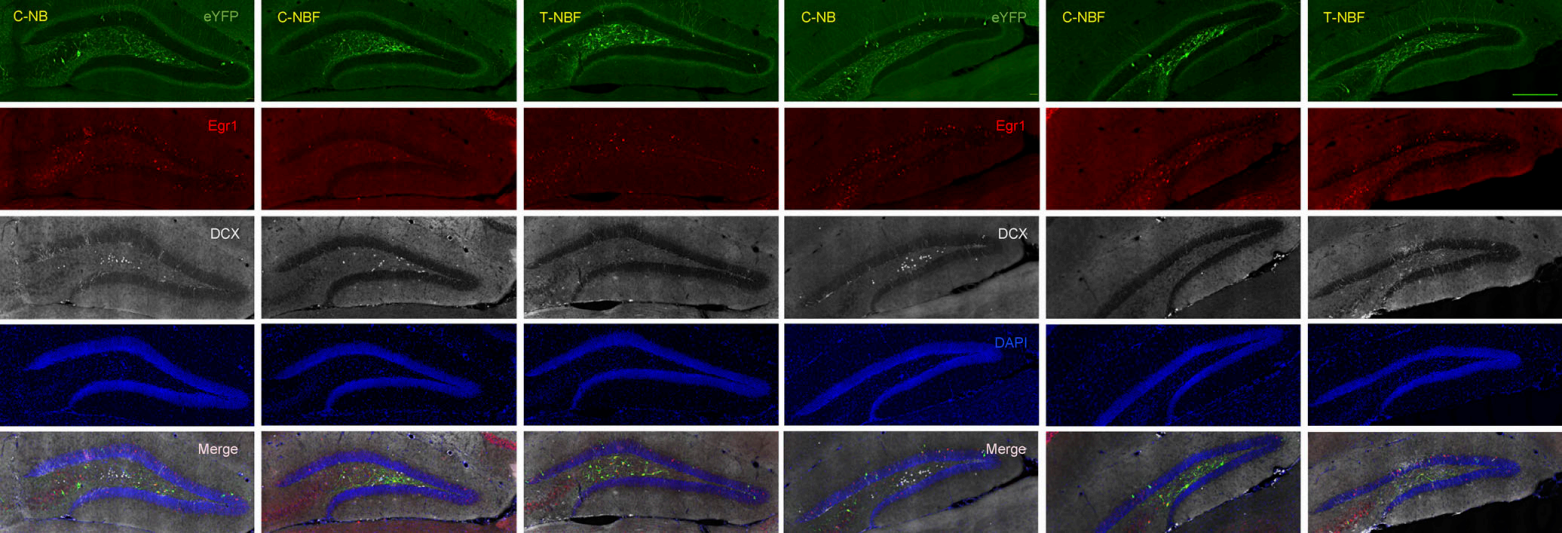
**Representative confocal images of whole DG taken from brain sections of the three experimental groups.** Immunohistochemistry of brain sections followed by confocal imaging captured engram cocktail–induced eYFP^+^ cells, Egr-1^+^ cells, immature neurons (DCX^+^), and their co-expression in the DG of C-NB, C-NBF, and T-NBF mice. Scale bar = 950 μm.

To further elucidate the role of new neurons in the engram, we next asked whether immature neurons that got recruited during contextual memory acquisition were reactivated upon the retrieval of this memory. To determine that, we examined the level of Egr-1 (Zif268), an immediate early gene previously implicated as a memory retrieval proxy (Guzowski et al., 2005; Hall et al., 2001; Radulovic et al., 1998). This analysis revealed that the total number of cells expressing the immediate early gene *Egr-1* following test-induced activation (Egr-1^+^ cells) was similar among the three groups, suggesting that the total number of neurons recruited during memory retrieval was not changed due to the FAD genotype or level of hippocampal neurogenesis (Fig. 3 A). Notably, the number of DCX^+^Egr-1^+^ cells was markedly reduced in C-NBF mice compared to C-NB mice (Fig. 3 B; *P = 0.0109). This number was significantly increased following augmentation of neurogenesis in the T-NBF mice, suggesting that increasing neurogenesis results in more immature neurons recruited during memory retrieval (Fig. 3 B; **P = 0.0058). The total number of eYFP^+^Egr-1^+^ cells recruited during both memory acquisition and retrieval was compromised in the C-NBF mice compared to the C-NB mice and increased in T-NBF mice (Fig. 3, C and H; *P = 0.0162, **P = 0.0037). The number of immature neurons recruited during memory and retrieval (DCX^+^eYFP^+^Egr-1^+^) revealed a reduced, albeit not statistically significant, number in C-NBF compared to C-NB, and a marked increase in the T-NBF group (Fig. 3, D and I; P = 0.0692, **P = 0.0015; Fig. S2). Notably, alterations in the number of immature neurons recruited during memory retrieval (DCX^+^eYFP^+^Egr-1^+^) between the three groups was directly related to their recruitment during acquisition. Evidently, the number of immature neurons that was activated during retrieval, but not during acquisition (DCX^+^eYFP^−^Egr-1^+^), revealed no effect of treatment or genotype (Fig. 3 E). This result suggests that augmenting neurogenesis specifically increases the number of new neurons that participate in memory acquisition. Of note, the number of mature neurons that were recruited during acquisition and reactivated during retrieval was comparable between the three experimental groups (Fig. 3 F). Examination of the ratio of immature and mature neurons participating in the neuronal ensemble in each of the experimental groups revealed that the number of immature neurons was reduced in the C-NBF compared to the C-NB with only 11% new neurons in the memory circuit in the DG of C-NBF compared to 20% in the C-NB. Following enhanced neurogenesis, this percentage increased to 35% in the T-NBF (Fig. 3 G). Taken together, these results suggest that the number of immature neurons that get recruited into the neuronal ensemble for memory formation is impaired in the FAD mice. As a result, the total number of neurons recruited into the engram is reduced in FAD, leading to diminished memory. Enhancement of neurogenesis resulted in an increased number of new neurons that participated in memory formation, resulting in the restoration of memory.

### Enhanced synaptic spine density of immature neurons participating in the engram in FAD mice following augmentation of neurogenesis

**Figure 4.**
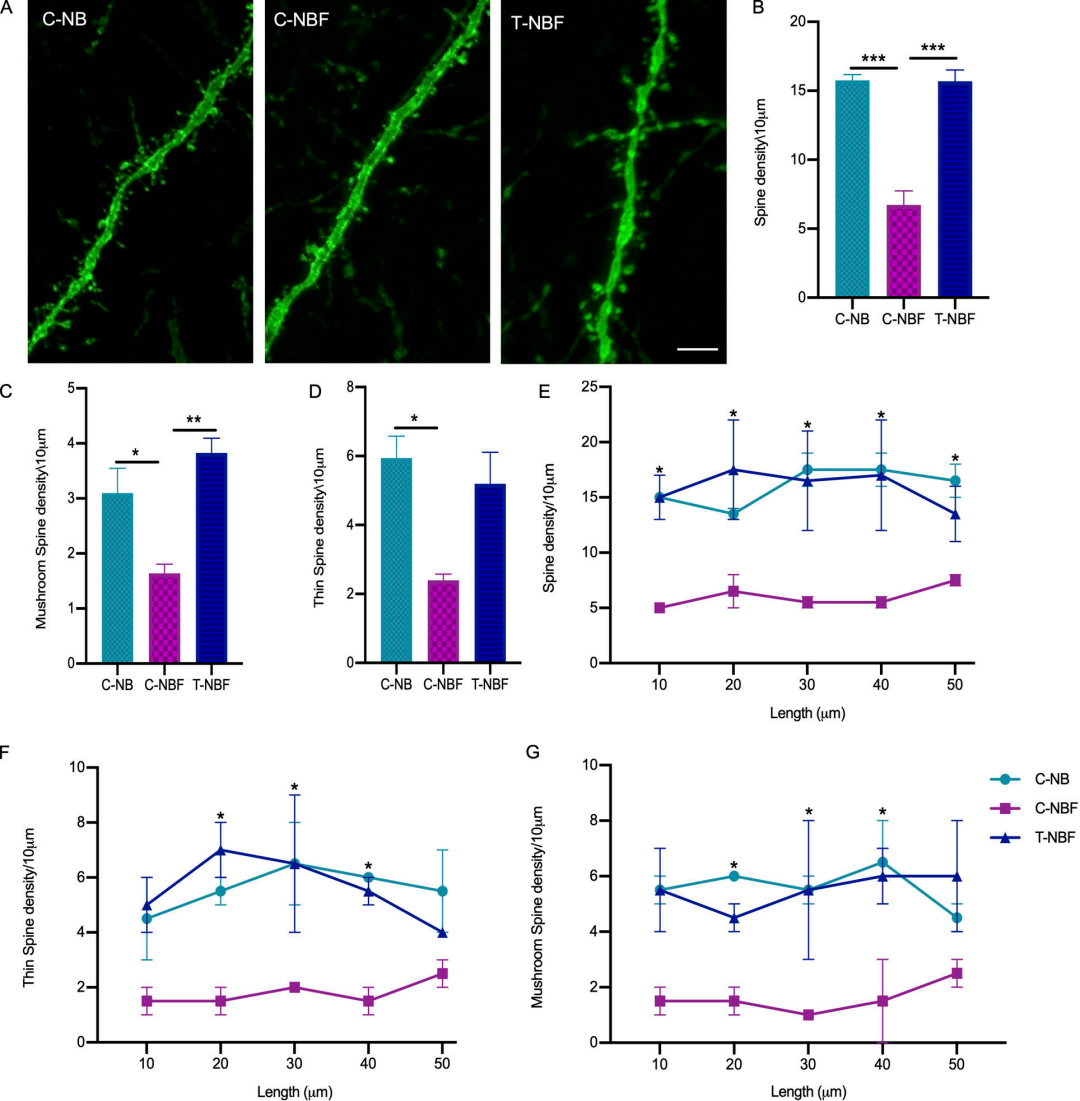
**Rescue of synaptic density in FAD mice following augmentation of neurogenesis. (A)** Confocal images showing dendritic spines in eYFP^+^Egr-1^+^DCX^+^ cells in brain sections of C-NB, C-NBF, and T-NBF mice. Scale bar = 25 μm. **(B–D)** The density of total (B; F(2, 6) = 43.02), mushroom (C; F(2, 6) = 12.46), and thin (D; F(2, 6) = 8.332) spines was quantified in 10 μm dendrite segments of eYFP^+^Egr-1^+^DCX^+^ engram cells in brain sections of C-NB, C-NBF, and T-NBF mice using Neurolucida 360. *N* = 3/group and *N* = 25/10 μm dendrite/animal. One-way ANOVA with Tukey’s multiple comparisons post hoc test, *P < 0.05, **P < 0.005, and ***P < 0.0005. **(E–G)** Total spine density (E; F(2, 15) = 28.87), thin spine density (F; F(2, 15) = 21.88), and mushroom spine density (G; F(2, 15) = 19.76) as a function of distance from the cell body. Two-way ANOVA with Tukey’s multiple comparisons post hoc test, *P < 0.05.

**Figure S3.**
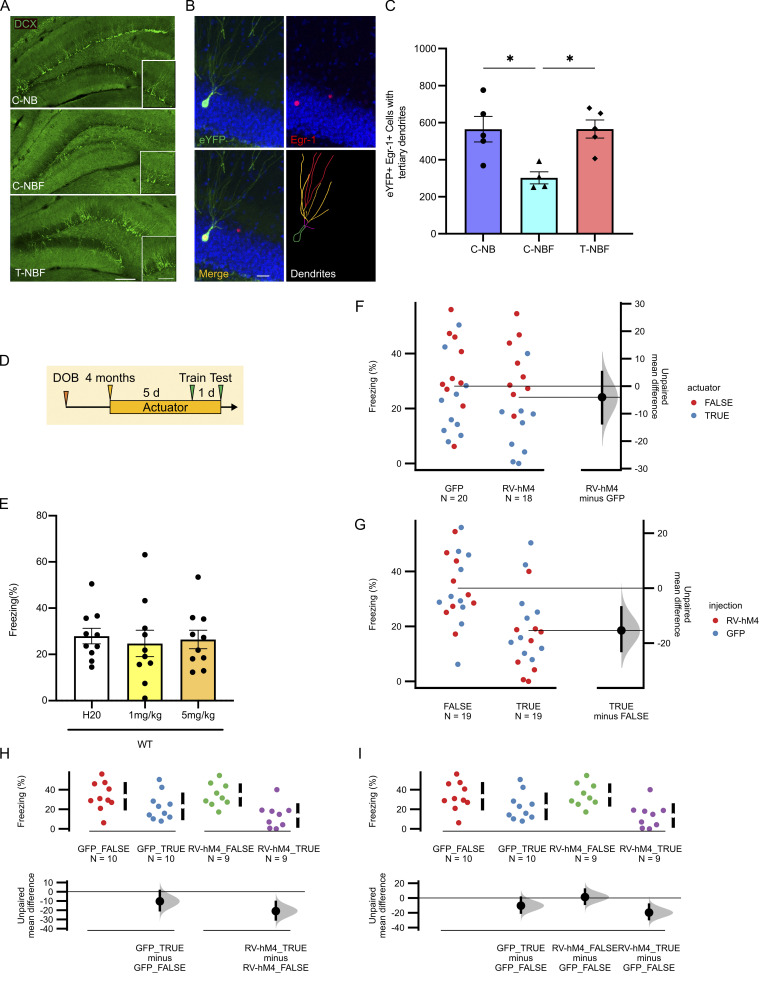
**Rescue of dendritic tree of engram cells following enhanced neurogenesis in FAD mice. (A)** Confocal images of DCX-expressing cells in the DG of the corn oil (C)–treated *NestinCreER*^*T2*^*; Bax*^*fl/fl*^ (NB), corn oil (C)– or tamoxifen (TAM)-treated *NestinCreER*^*T2*^*; Bax*^*fl/fl*^*;* 5XFAD (NBF). Scale bar = 100 μm; insert = 50 μm. **(B)** Confocal images of tertiary dendrites of neurons incorporated in the contextual memory circuit. Scale bar = 20 μm. **(C)** Quantification of tertiary dendrites in engram cells, as observed in the outer molecular layer of the DG (*P < 0.05). **(D–I)** Validation of actuator and injection effect on freezing behavior. **(D**) Experimental paradigm. **(E)** Treatment of wild type mice with either vehicle (H_2_O), 1 mg/kg CNO, or 5 mg/kg CNO results in comparable freezing behavior, suggesting lack of effect of CNO on the behavioral phenotype (*N* = 10; one-way ANOVA with Fisher’s LSD post hoc test F(2, 27) = 0.1289, P = 0.8796, ns). **(F)** Gardner-Altman plot of actuator. **(G)** Gardner-Altman plot of injection site. **(H)** Two group plot. **(I)** Cummings estimation plot. For F–I, colored points represent raw data, black points represent mean difference between condition compared to reference group (indicated by the gray horizontal line), and black vertical lines with distribution represent the bias-corrected and accelerated 95% confidence interval after performing bootstrap resampling 5,000 times. For H and I, vertical black lines in the top row next to colored points represent standard deviation; gaps between vertical black lines represent the mean (Ho et al., 2019).

Synaptic pathology is one of the earliest impairments in AD and correlates with memory deficits (Hsia et al., 1999; Jacobsen et al., 2006; Terry et al., 1991). To elucidate whether enhanced recruitment of new neurons into the engram was linked to restoration of memory following augmentation of neurogenesis in the T-NBF mice, we asked whether there was a change in spine density of these cells. To answer this, we quantified the density of dendritic spines in DCX^+^eYFP^+^Egr-1^+^ cells in the DG of the C-NB, C-NBF, and T-NBF mice. Consistent with the notion that dendritic spines, which are important in memory formation, are impaired in AD (Jacobsen et al., 2006; Roy et al., 2016; Terry et al., 1991), we observed that new neurons recruited to the engram in C-NBF exhibited reduced synaptic density compared to C-NB (Fig. 4, A and B). Notably, the synaptic density of engram new neurons was restored in T-NBF (Fig. 4, A and B). To gain an insight into the type of spines observed in dendrites of engram cells, we quantified the number of mushrooms and thin spine densities. We observed that the number of mushroom spines was deficient in dendrites of C-NBF mice compared to C-NB and restored in T-NBF mice (Fig. 4 C). A similar trend was observed in thin spines; however, the difference between C-NBF and T-NBF was not statistically significant (Fig. 4 D). Quantification of spine density as a function of distance from the cell body revealed comparable density in the C-NB and T-NBF, while in the C-NBF spine density was consistently and significantly low (Fig. 4, E–G). Interestingly, we observed that the augmentation of neurogenesis rescued the number of tertiary dendrites in the engram (eYFP^+^Egr-1^+^ cells; Fig. S3, A–C). Together, these results support a role for new neurons in memory restoration in the T-NBF mice.

### Augmentation of neurogenesis restores synaptic spine density of mature granule neurons participating in the engram in FAD

**Figure 5.**
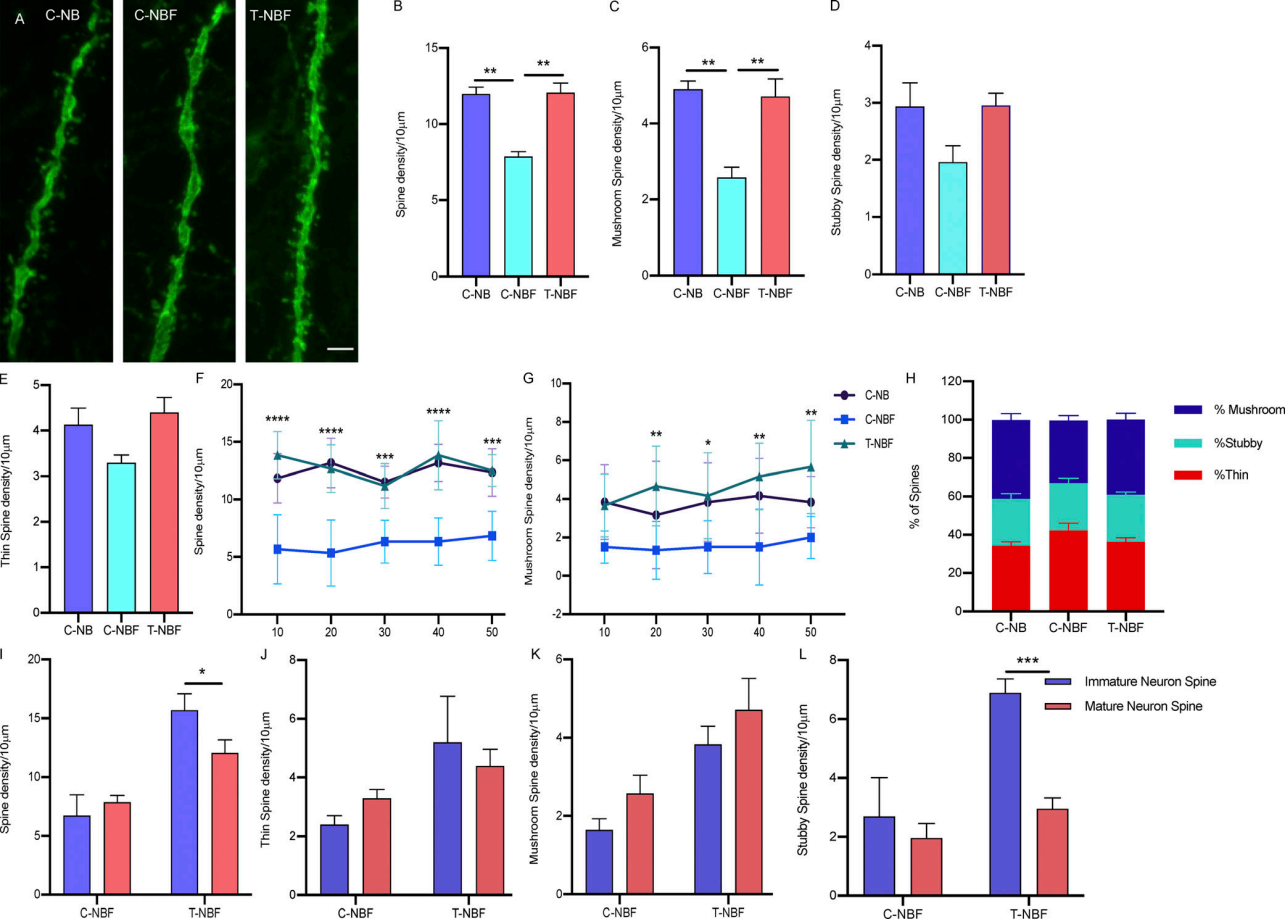
**Augmentation of neurogenesis modulates synaptic plasticity of mature granule neurons participating in the engram in the DG. (A)** Confocal images showing dendritic spines of mature neurons (eYFP^+^Egr1^+^NeuN^+^) in the DG of C-NB, C-NBF, and T-NBF. **(B–E)** Total spine density (B; F(2, 6) = 24.31), mushroom (C; F(2, 6) = 15.07), stubby (D; F(2, 6) = 3.228), and thin (E; F(2, 6) = 3.713) spines were quantified in 10 μm dendrite segments of eYFP^+^Egr1^+^NeuN^+^ engram cells in DG using Neurolucida 360. *N* = 3/group and *N* = 50/10 μm dendrite/animal were analyzed. **(F and G)** Total spine density (F; F(2, 75) = 90.06) and mushroom spine density (G; F(2, 75) = 22.04) as a function of distance from the cell body. Two-way ANOVA with Tukey’s multiple comparisons post hoc test, *P < 0.05, **P < 0.005, and ***P < 0.0005. **(H) **Percentage of each type of spines/10 μm. F(2, 18) = 22.59. **(I–L) **Comparison of total (I; F(1, 8) = 10.24), thin (J; F(1, 8) = 0.009), mushroom (K; F(1, 8) = 0.0068), and stubby (L; F(1, 8) = 27.82) spine density in mature and immature engram neurons in the DG of C-NBF versus T-NBF. Two-way ANOVA with Bonferroni’s multiple comparisons post hoc test, *P < 0.05, **P < 0.005, and ***P < 0.0005.

In light of the effect of augmented neurogenesis on the spine density of immature neurons participating in the engram, we asked whether this process affects the spine density of mature granule neurons in the DG that play a role in the engram. To examine this, we quantified the density and morphology of dendritic spines of NeuN^+^eYFP^+^Egr-1^+^ in the granular cell layer in brain sections of C-NB, C-NBF, and T-NBF mice (Fig. 5 A). Quantification of spine density revealed that the spine density of NeuN^+^eYFP^+^Egr-1^+^ neurons in the DG of C-NBF was reduced compared to C-NB mice (Fig. 5 B). Interestingly, we observed that the spine density of NeuN^+^eYFP^+^Egr-1^+^ neurons in the DG of T-NBF mice was comparable to that in the C-NB mice (Fig. 5 B). This result suggests that augmenting neurogenesis restores synaptic plasticity non-autonomously in mature neurons in the DG. To examine whether particular forms of dendritic spines are modulated in the engram, we quantified the density of mushroom, stubby, and thin spines. The data showed that the density of mushroom spines was significantly deficient in NeuN^+^eYFP^+^Egr-1^+^ neurons in C-NBF mice (Fig. 5 C). The density of mushroom spines was restored in the T-NBF mice (Fig. 5 C). The density of stubby and thin spines showed similar trends, albeit not statistically significant (Fig. 5, D and E). Examination of spine density as a function of distance from the cell body revealed consistent impairment in total spine density in neurons in the C-NBF mice independently of the distance from the cell body compared to both C-NB and T-NBF (Fig. 5 F). Deficits in mushroom spine density in C-NBF were more pronounced at a distance >10 μm from the cell body in C-NBF, compared to C-NB and T-NBF (Fig. 5 G). Examination of the ratio of spine types in granule neurons in the three groups revealed that the majority of spines in granule neurons in the C-NB and T-NBF mice are mushroom (∼40%), while in C-NBF the majority are thin spines (Fig. 5 H). We next compared the change in spine density of immature versus mature neurons following augmentation of neurogenesis in T-NBF. The data showed that while spine density has increased in both immature and mature neurons in T-NBF mice compared to C-NBF, the overall spine density in immature neurons was greater than that in mature neurons (Fig. 5 I). Interestingly, while all three spine types increased in both mature and immature neurons in T-NBF compared to C-NBF (Fig. 5, J–L), the density of stubby spines significantly increased in immature but not in mature neurons in the T-NBF mice compared to the C-NBF (Fig. 5 L). Taken together, these results suggest that augmenting neurogenesis in FAD restores spine density deficits in mature granule neurons in the DG.

### Immature neurons are required for proper memory formation

**Figure 6.**
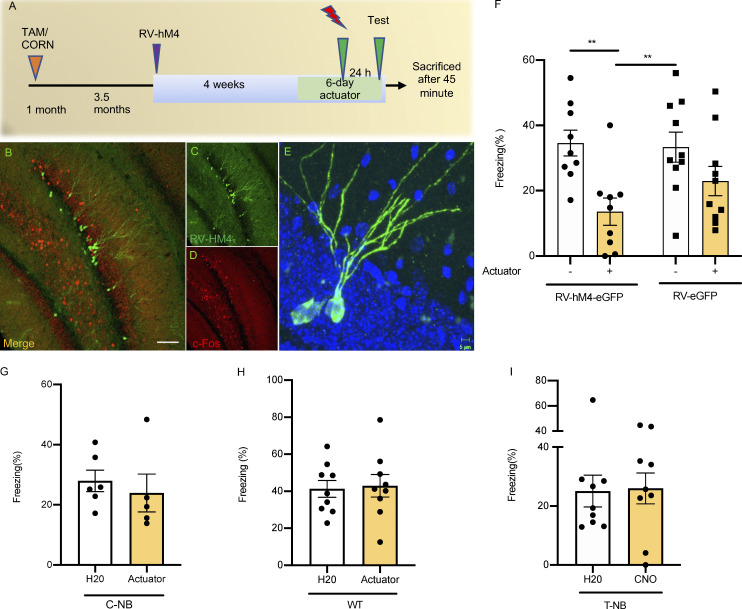
**New neurons are required for the formation of CFC memory in FAD. (A)** Experimental design aiming at determining level of mouse freezing following actuator-induced inactivation of new neurons infected with RV-HM4-eGFP. **(B–D)** Confocal images of RV-HM4-eGFP–infected new neurons and c-fos^+^ cells in brain sections of tamoxifen-treated *NestinCreER*^*T2*^*; Bax*^*fl/fl*^*;* 5XFAD (T-NBF; merged image [B], RV-HM4-GFP [C], and c-fos [D]). Scale bar = 100 μm. **(E)** Representative image of HM4-GFP^+^ neuron in the granular cell layer of tamoxifen-treated *NestinCreER*^*T2*^*; Bax*^*fl/fl*^*; *5XFAD (T-NBF). Scale bar = 5 μm. **(F)** Tamoxifen-treated *NestinCreER*^*T2*^*; Bax*^*fl/fl*^*;* 5XFAD (T-NBF) injected with RV-HM4-eGFP or RV-eGFP followed with actuator (+) or not (−) exhibit compromised memory compared to vehicle-treated (two-way ANOVA with Fisher’s LSD post hoc test for virus type: F(1, 34) = 0.8621, P = 0.3597; and for actuator: F(1, 34) = 12.99, **P = 0.0021, **P = 0.0029, respectively). **(G–I)** RV-HM4-eGFP-injected C-NB (G), wild type C57Bl6 (H), and T-NB (I) treated with CNO or water during CFC show similar behavior. Student’s two-tailed *t* test, P = 0.310 (G); Student’s two-tailed *t* test, P = 0.829 (H); Student’s two-tailed *t* test, P = 0.906 (I).

In light of these results, we asked whether immature neurons in T-NBF mice were necessary for the rescue of memory observed in these mice. To address this, T-NBF mice were injected with retroviral vectors expressing the Gi Designer Receptors Exclusively Activated by Designer Drugs (DREADD) RV-hM4Di-eGFP 4 wk before CFC to specifically inactivate the newly mature neurons (Fig. 6 A). To validate that RV-hM4Di-eGFP inactivates infected cells, mice injected with RV-hM4Di-eGFP were treated with clozapine N-oxide (CNO) and their brain sections were examined for the expression of eGFP and endogenous c-fos. We observed no overlap between the expression of eGFP and endogenous c-fos, confirming that RV-hM4Di-eGFP inactivates infected cells (Fig. 6, B–E). To further validate the specificity of DREADD in our mice, we examined a putative side effect of actuator treatment in mice that were not injected with the Gi-DREADD receptor. We observed no change in their freezing level (Fig. S3, D and E) or the effect of the actuator or injection site (Fig. S3, F–I). Next, we examined the effect of inactivating immature neurons on memory formation in CFC in T-NBF mice. For this, T-NBF mice were stereotaxically injected with either RV-hM4Di-eGFP or control virus (RV-eGFP) 4 wk before CFC. 5 d before the behavioral test, mice were treated with either actuator or water (Fig. 6 A). Treatment of RV-hM4Di-eGFP-injected T-NBF mice with actuator significantly diminished memory in these mice (Fig. 6 F; two-way ANOVA with Fisher’s LSD post hoc test for virus type: F(1, 34) = 0.8621; P = 0.3597 and for actuator: F(1, 34) = 12.99; P = 0.0010 and **P = 0.0021, **P = 0.0029, respectively). Of note, inhibiting the activity of new neurons during CFC in RV-hM4Di-eGFP-injected wild-type mice, C-NB, or T-NB mice did not affect mouse performance (Fig. 6, G–I). The results in wild-type and C-NB mice support previous observations in thymidine kinase–expressing mice showing that the deletion of new neurons does not compromise performance in CFC (Deng et al., 2009; Hollands et al., 2017). The lack of effect in T-NB mice aligns with the observation that C-NB and T-NB mice exhibited similar performance in the CFC task and suggests that the additional immature neurons in the T-NB mice were not essential for functional CFC memory engram, supporting our hypothesis of a ceiling effect in these mice. Taken together, these results show that more immature neurons are required for proper memory formation in the T-NBF mice, and their deficiency in C-NBF underlies memory impairments. Increasing their numbers in the T-NBF mice rescued memory, and in turn, their inactivation disrupted engram and memory formation. In summary, augmenting neurogenesis in FAD mice increased the availability of new neurons to be incorporated in the contextual memory engram, resulting in proper engram formation and intact performance in memory tasks. In addition, immature neurons are favorably reactivated during memory retrieval and are required for the rescue of contextual memory in FAD. Notably, we show that in addition to immature neurons, augmenting neurogenesis affects synaptic plasticity in mature granule neurons participating in the engram.

### Augmenting neurogenesis in FAD leads to an engram transcription profile that resembles the wild type

**Figure 7.**
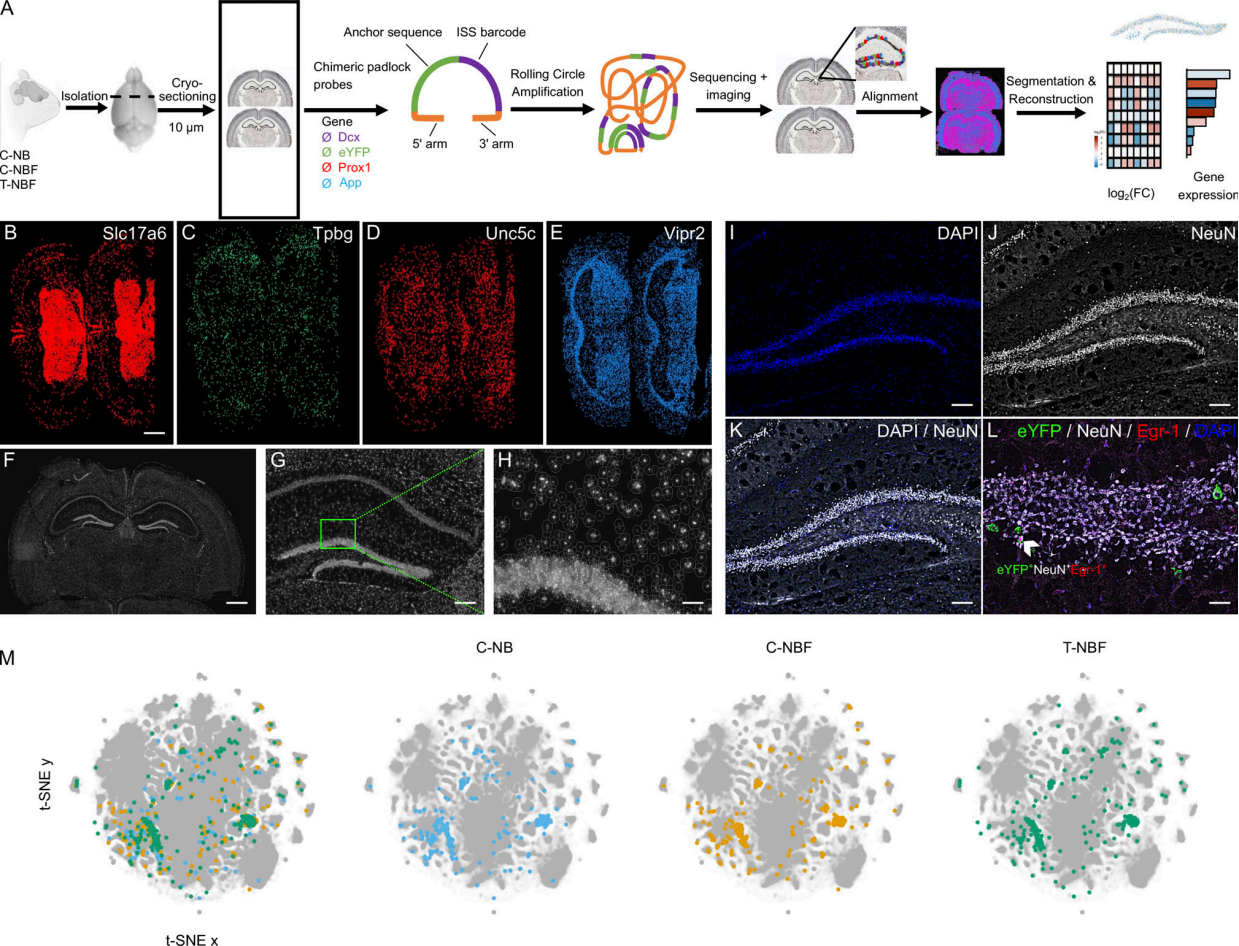
**In situ sequencing of immature and mature engram neurons. (A)** A scheme of the in situ sequencing workflow. **(B–E)** Representative images of individual gene expression readout following sequencing and imaging. *Slc17a6* (B), *Tpbg* (C), *Unc5c* (D), and *Vipr2* (E). Scale conversion for individual images = 0.32 μm/pixel. **(F–H)** Cell segmentation. An image of DAPI-stained nuclei is shown with the estimated cell border boundary overlaid. **(I–L)** Representative example of DAPI (I), NeuN (J), eYFP^+^NeuN^+^Egr-1^+^DAPI^+^ neuron (L, white arrowhead)–stained cells that underwent in situ sequencing. **(M)** *t*-SNE plots of the in situ sequencing data. Points represent 50 μm^2^–binned areas covering the entire section. Areas in the DG for C-NB, C-NBF, and T-NBF are shown in blue, orange, and green, respectively, above other areas (gray). Scale bar = 1,000 μm (B–F); 225 μm (G); 55 μm (H); 175 μm (I–K); 75 μm (L).

**Figure S4.**
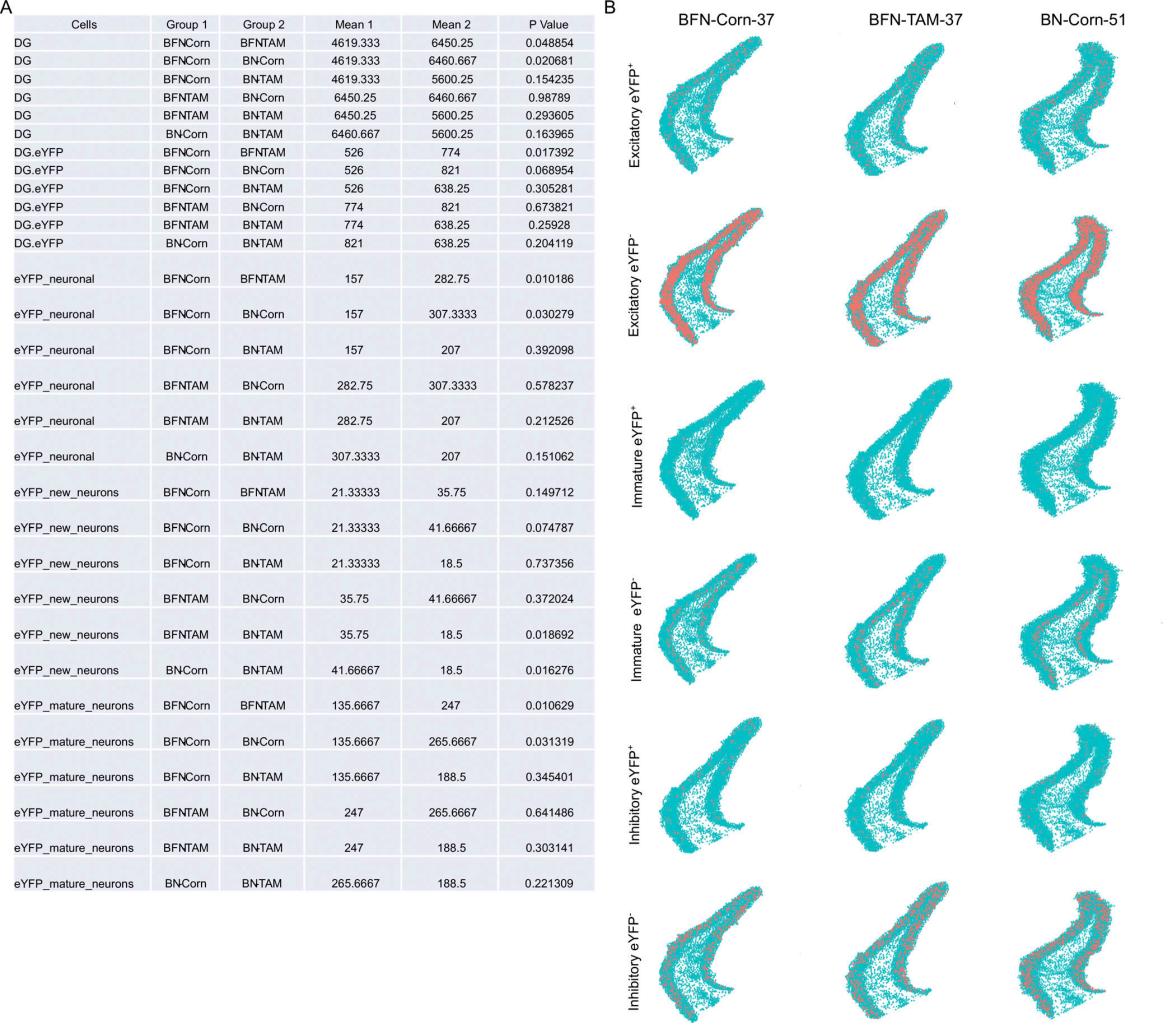
**Quantification and distribution of neurons in the dentate gyrus based on in situ sequencing data. (A)** Quantification of the number of *eYFP*^+^ new, mature and combined neurons in the DG of the three experimental groups, C-NB, C-NBF, and T-NBF, based on in situ sequencing data. *t* test analysis. **(B)** Plots showing the distribution of all *eYFP*^+^, *eYFP*^−^ excitatory, inhibitory, immature, and mature neurons in the DG.

**Figure 8.**
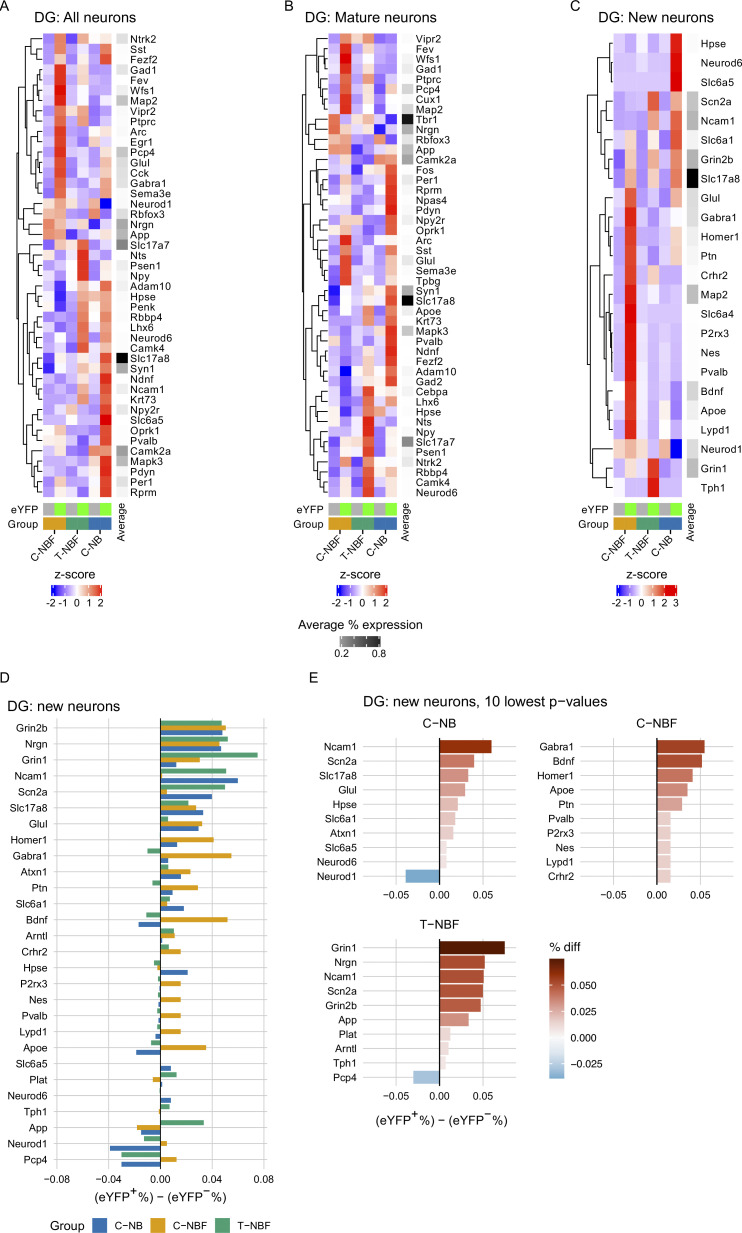
**Altered engram profile in FAD is rescued following augmentation of neurogenesis. (A–C)** Heatmap analysis of genes differentially expressed in *eYFP*^+^ compared to *eYFP*^−^ total neurons (A), mature neurons (B), and new neurons (C) in the DG of C-NB, C-NBF, and T-NBF mice. Plotted values are *z*-scored % expression. Gray scale plots the mean expression over all data sets for each gene. Only genes with P < 0.1 (nominal P value) for at least one of the *eYFP*^+^ versus *eYFP*^−^ comparisons were retained. *eYFP*^−^ columns are indicated by gray box below heatmap; *eYFP*^+^ columns indicated by yellow-green box. C-NB: blue; C-NBF: orange; T-NBF: green. **(D)** Intergroup directionality of the genes with the lowest P values for percent expression [(*eYFP*^+^ %) − (*eYFP*^−^ %)] in new neurons. **(E)** The genes with the 10 lowest P values in newborn neurons for each group, where the bar indicates the difference in percent expression [(*eYFP*^+^ %) − (*eYFP*^−^ %)].

The results thus far suggest that augmentation of neurogenesis modulated hippocampal function in FAD (T-NBF) mice. Therefore, we sought to examine the signaling pathways of the engram in FAD compared to wild-type mice, and their alterations following augmentation of neurogenesis. In addition, we examined whether augmentation of neurogenesis affects the profile of the granule cells in the DG. Thus, we examined the transcription signature of immature and mature neurons recruited into the engram in the DG of C-NB, C-NBF, and T-NBF mice. For this purpose, C-NB, C-NBF, and T-NBF mice were injected with the engram cocktail, placed on a doxycycline diet, and subjected to CFC, as before. Mice were sacrificed 45 min after the test phase of CFC and brains were cryosectioned. Coronal sections were analyzed by spatial transcriptomics (Fig. 7 A). 159 genes of interest were sequenced simultaneously with single-cell resolution using in situ sequencing (Fig. 7, A–E). To assign reads to individual cells, images of DAPI nuclei-stained brain sections were cell segmented using a custom MATLAB script (Fig. 7, F–L). Quantification of the number of cells revealed that the average total cell count in brain sections was comparable among the three groups (C-NB: 152,492 ± 8,000; C-NBF: 130,761 ± 2,200; T-NBF: 145,158 ± 6,000). Interestingly, the total cell count in the DG revealed 6,460 ± 235 cells ± SE in the C-NB group, 4,619 ± 325 in the C-NBF group, and 6,450 ± 313 in the T-NBF group (C-NBF versus T-NBF *t* test, P = 0.048; C-NB versus C-NBF, P = 0.02; C-NB versus T-NBF, P = 0.98; Fig. S4, A and B). This may suggest that the number of cells in the DG depends on the level of neurogenesis in these mice. To identify engram cells in the DG, we examined the expression of *eYFP* in a uniformly traced area of the DG that included the hilus, subgranular, and the granular layers of the DG. Cells were defined as *eYFP*^+^ if they contained at least one *eYFP* read. Fisher’s exact test (FET) was used to compare the proportion of cells expressing each gene across pairwise groups for each cell type. Fig. 7 M shows *t*-distributed stochastic neighbor embedding (*t*-SNE) plots of the expression pattern in the DG of the three experimental groups. To gain an insight into the molecular profile of engram neurons, we compared the profile of *eYFP*^+^ neurons with one of the *eYFP*^−^ neurons in the C-NB group. An array of genes critical for neuronal function, such as *Syn1* (synapsin 1), *Ndnf* (neuron-derived neurotrophic factor), *Ncam1* (neural cell adhesion molecule), *Npy2r* (neuropeptide Y receptor Y2), *Slc6a5* (solute carrier family 6 member 5), *Oprk1* (opioid receptor kappa 1), *Mapk3* (mitogen-activated protein kinase 3), and *Gabra1* (gamma aminobutyric acid type A receptor subunit alpha 1), was upregulated in the *eYFP*^+^ neurons compared to *eYFP*^−^ (Fig. 8 A). Interestingly, several AD-linked genes, such as *App*, *Adam10*, and *Psen1*, were upregulated as well (Fig. 8 A). Some genes, such as *App* (amyloid precursor protein), *Fos*, *Npas4* (neuronal pas protein 4), *Npy2r*, *Oprk1*, *Sst* (somatostatin), *Glul* (glutamate ammonia ligase), *Syn1*, *Slc17a8* (solute carrier family 17 member 8), *ApoE* (apolipoprotein E), *Mapk3*, *Adam10* (A disintegrin and metalloproteinase domain-containing 10), *Pvalb* (parvalbumin), and *Gad2* (glutamate decarboxylase 2), were upregulated in *eYFP*^+^ mature neurons compared to *eYFP*^−^ mature neurons in the C-NB mice (Fig. 8 B). In *eYFP*^+^ new neurons, genes such as *Neurod6* (neuronal differentiation 6), *Slc6a1*, *Slc6a5*, *Slc17a8*, *Ncam1* (neural cell adhesion molecule 1), and *Grin2b* (glutamate receptor, ionotropic, N-methyl D-aspartate 2B) were upregulated compared to *eYFP*^−^ new neurons (Fig. 8 C). Interestingly, transcript profile of *eYFP*^+^ neurons in C-NBF mice was the opposite to the one in C-NB (Fig. 8 A). For the most part, in the engram in C-NBF, there was no major change in the expression of genes that were vastly upregulated in the C-NB engram (Fig. 8 A), while a separate set of genes, not greatly modulated in the C-NB, such as *Fev*, *Wfs1*, *Map2*, *Vipr2*, *Ptprc*, *Arc*, *Egr-1*, was upregulated in the C-NBF engram (Fig. 8 A). Notably, the profile of engram neurons in T-NBF partially mirrored the one of the C-NB (Fig. 8 A). Examination of the profile of mature and new neurons in the engram in the different groups revealed similar trends (Fig. 8, B and C). The profile of *eYFP*^+^ mature neurons compared to *eYFP*^*−*^ mature neurons in C-NBF was, to a great extent, the opposite of their profile in C-NB mice (Fig. 8 B). The expression profile in the T-NBF group partially resembled each of the other groups (Fig. 8 B). Similar to the expression profile of *eYFP*^+^ mature neurons, the profile of *eYFP*^+^ new neurons in C-NBF was the opposite of the one in C-NB (Fig. 8 C). Interestingly, the gene expression of *eYFP*^+^ new neurons in the T-NBF resembled the profile of these cells in the C-NB group (Fig. 8, C and D). Genes regulating neuronal function, such as *Ncam1*, had the lowest P value in (*eYFP*^+^/*eYFP*^−^) new neurons in both C-NB and T-NBF mice (Fig. 8 E). Taken together, these results show that neurons recruited into the engram have a distinct gene expression profile compared to the rest of their peers. This profile is vastly different in the FAD mice and is partially restored following augmentation of neurogenesis. Particularly faithful restoration of the engram profile was apparent in new neurons and total neurons following enhancement of neurogenesis.

### AD-linked signals modulate the engram

**Figure 9.**
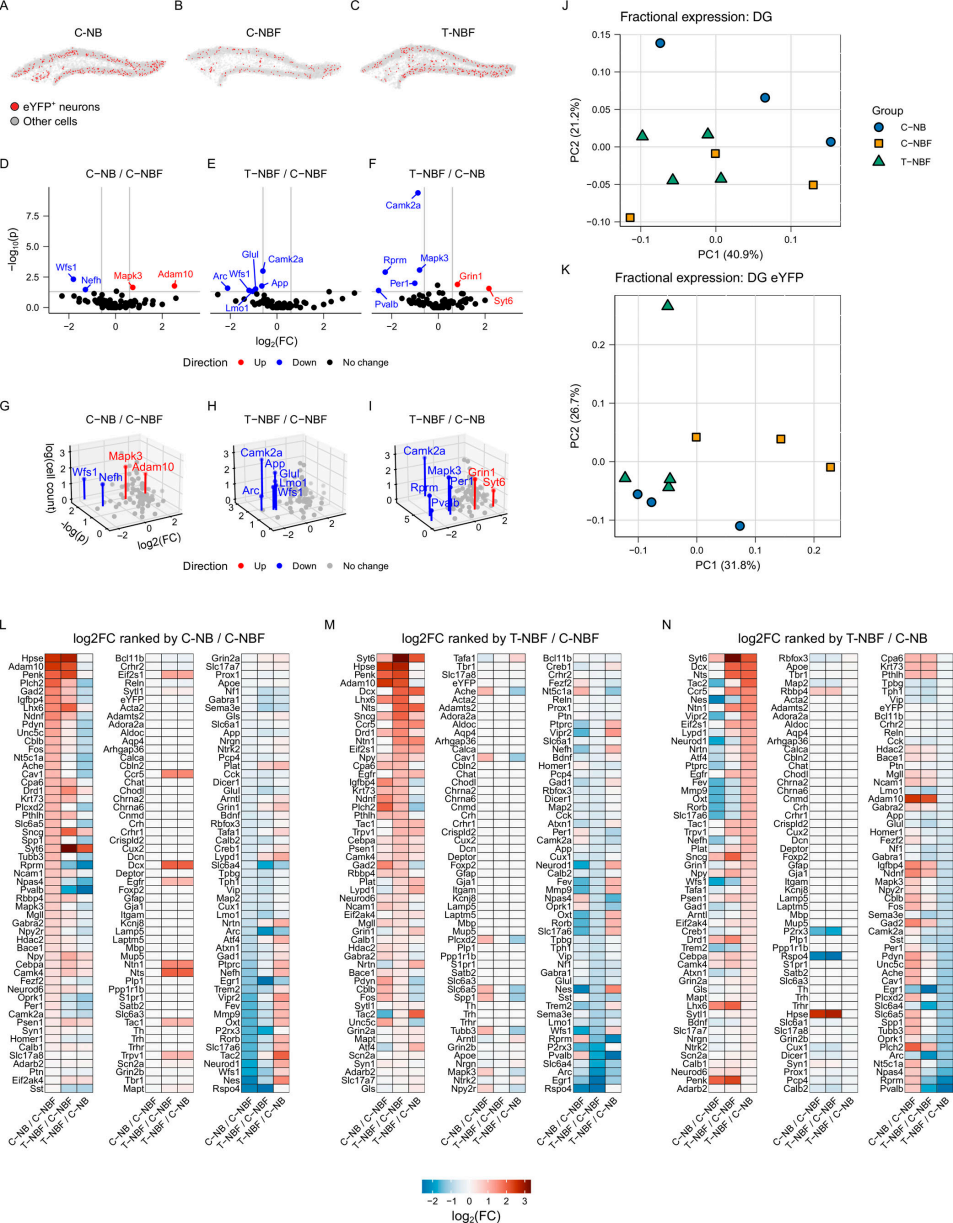
**Transcript profile of the engram in FAD and following augmentation of neurogenesis. (A–C)** Representative scatter plots showing *eYFP*^+^ neurons located within the DG and hilus for C-NB (A), C-NBF (B), and T-NBF (C) mice. Red points indicate *eYFP*^+^ neurons, and gray points indicate all other cells. **(D–F)** 2D volcano plots for each comparison. For 2D volcanos: Log_2_FC vs. −log_10_(P) for C-NB/C-NBF (D), T-NBF/C-NBF (E), and T-NBF/C-NB (F). **(G–I)** 3D volcano plots for each comparison: C-NB/C-NBF (G), T-NBF/C-NBF (H), and T-NBF/C-NB (I). X axis: log_2_FC; y axis: −log(P value); z axis: log(cell count) = log[(# positive cells in group 1) + (# positive cells in group 2)]. **(J and K)** ANOSIM. Principal component analysis plots of fractional gene expression of all DG neurons (J) and of eYFP^+^ neurons (K) in the three experimental groups (for statistical analysis, see Fig. S4). **(L–N)** Heatmaps representing the log_2_FC of the percentage of *eYFP*^+^ neurons expressing each gene for C-NB/C-NBF log_2_FC, and T-NBF/C-NBF log_2_FC, and T-NBF/C-NB log_2_FC, ranked by the log_2_FC for C-NB/C-NBF (L), T-NBF/C-NBF (M), and T-NBF/C-NB (N).

**Figure S5.**
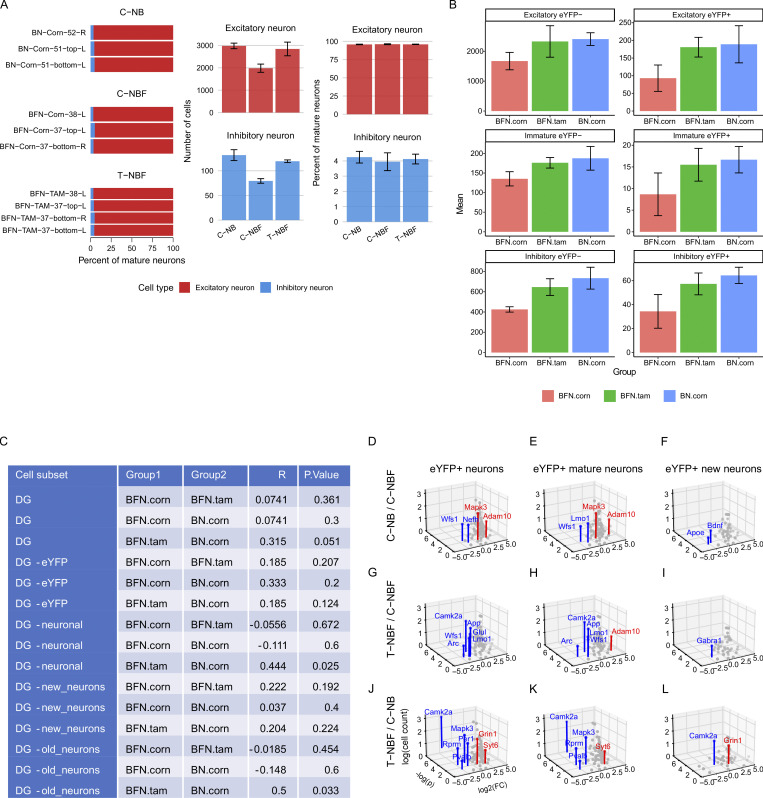
**Excitatory and inhibitory neurons in the granule cell layer of the DG. (A)** Excitatory and inhibitory percentage of mature neurons for each DG (left) and mean excitatory and inhibitory neurons (middle) and percent of total mature neurons (right) for each group. **(B)** Mean number of neurons for each group by excitatory/inhibitory, immature/mature, and eYFP^+/−^. **(C)** ANOSIM of fractional gene expression for each cell type between groups. **(D–L)** 3D volcano plots for eYFP^+^ total, mature, and immature neurons for each comparison. X axis: log_2_FC; y axis: negative log(P); z-axis: log(cell count).

Next, we compared the profile of the engram between the three experimental groups, regardless of the profile of the rest of the neurons in the DG that were not recruited into the engram. Examination of the distribution of *eYFP*^+^ neurons located within the DG and hilus revealed a reduced distribution of *eYFP*^+^ neurons in C-NBF compared to C-NB and T-NBF (Fig. 9, A–C; and Fig. S4, A and B). Differential gene expression in the total engram population, i.e., *eYFP*^+^ neurons in C-NB compared to C-NBF (Fig. 9, D and G) revealed that *Mapk3* and *Adam10* were the most upregulated genes in C-NB relative to C-NBF (>1.5 FC, uncorrected P < 0.05; Fig. 9, D and G). *Wfs1* (wolframin ER transmembrane glycoprotein) and *Nefh* (neurofilament heavy chain) were most downregulated in C-NB relative to C-NBF (>1.5 FC, uncorrected P < 0.05; Fig. 9, D and G). *Camk2a* (calcium/calmodulin-dependent protein kinase II alpha), *App*, *Glul*, *Wfs1*, *Arc* (activity-regulated cytoskeleton associated protein), and *Lmo1* (LIM domain only 1) were the most downregulated in T-NBF relative to C-NBF (Fig. 9, E and H). *Grin1* (glutamate ionotropic receptor NMDA type subunit 1) and *Syt6* (synaptotagmin 6) were most upregulated in T-NBF relative to C-NB, while *Camk2a*, *Mapk3*, *Rprm* (Reprimo), *Per1* (period circadian regulator 1), and *Pvalb* were most downregulated (Fig. 9, F and I). Fractional expression in the total DG revealed random order of the experimental groups (Fig. 9 J and Fig. 5 C). Interestingly, fractional expression of *eYFP*^+^ cells revealed a close expression pattern between T-NBF and C-NB groups, while C-NBF exhibited a distinct expression pattern (Fig. 9 K and Fig. 5 C). All 159 genes are listed in Fig. 9, L–N, and ranked by log_2_FC of the C-NB/C-NBF (Fig. 9 L), T-NBF/C-NBF (Fig. 9 M), and T-NBF/C-NB (Fig. 9 N) comparisons, respectively. Next, we attempted to examine whether augmentation of neurogenesis has affected the ratio of excitatory: inhibitory neurons in the DG. For this purpose, we quantified the number of mature and immature neurons that express genes known to regulate either inhibitory or excitatory activity (see Materials and Methods for the full list of genes). We observed that the ratio of inhibitory: excitatory was similar in the three experimental groups (Fig. S5 A). We next examined the distribution of immature and mature neurons in these subpopulations, as well as whether these neurons play a role in the engram (*eYFP*^+^). The data showed that augmenting neurogenesis contributed mostly to excitatory immature and mature neurons (Fig. S5 B). In addition, the number of inhibitory *eYFP*^−^ mature neurons was higher in the T-BFN and C-BN compared to C-NBF (Fig. S5 B). However, it should be noted that all neurons defined as inhibitory expressed at least one excitatory proxy. Thus, further experiments will need to validate the function of these neurons.

**Figure 10.**
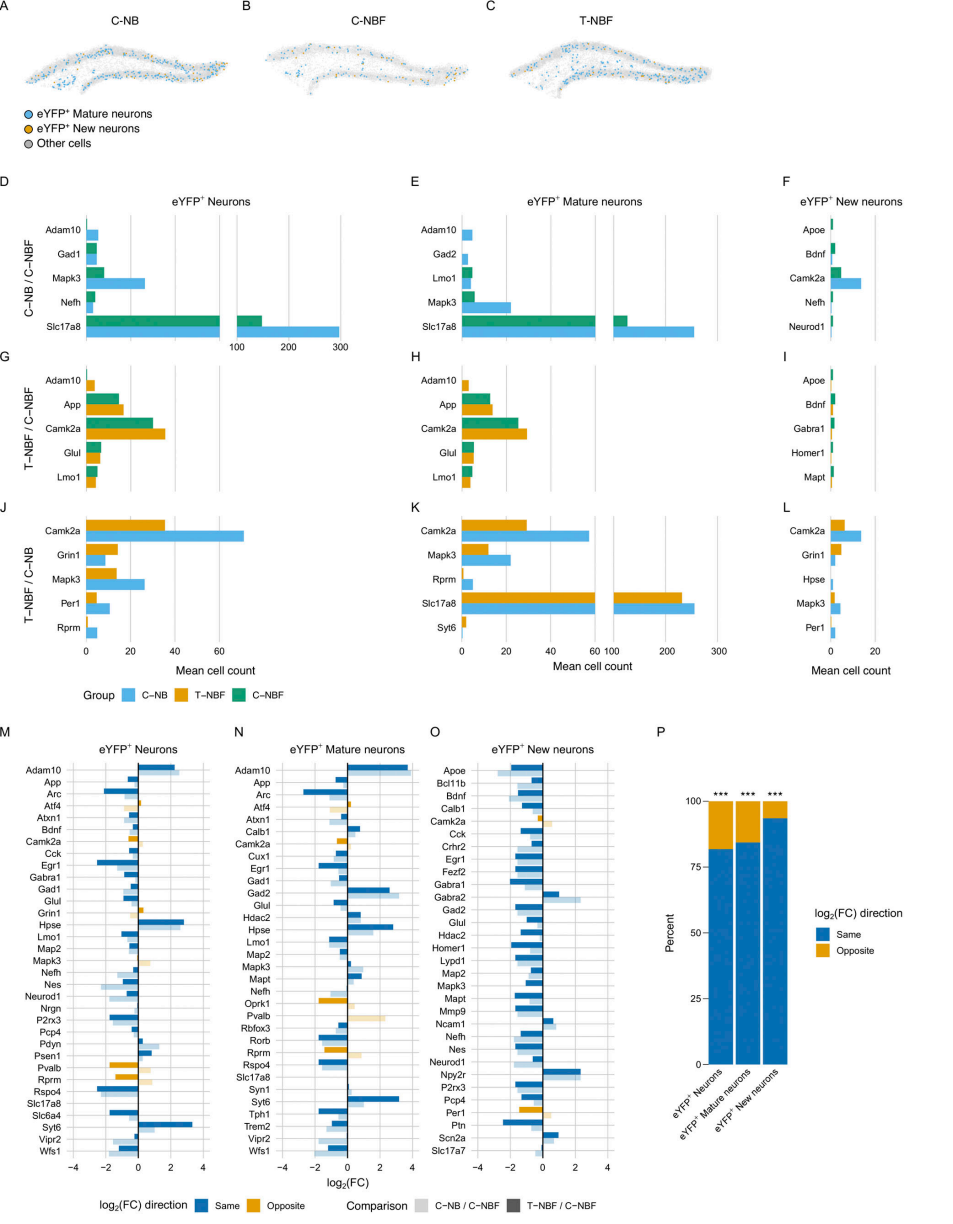
**Alteration of transcription profile of new and mature engram neurons in the DG of FAD following enhanced neurogenesis. (A–C)** Representative scatter plots of *eYFP*^+^ new and mature engram neurons in the DG of C-NB (A), C-NBF (B), and T-NBF (C) groups. Blue: *eYFP*^+^ mature neurons; orange: *eYFP*^+^ new neurons; gray: other cells. **(D–L)** Mean cell count of the five genes with the lowest P values for each comparison and cell type. Blue: C-NB; orange: T-NBF; green: C-NBF. **(M–O)** Consistency of log_2_FC direction for the union of the 20 genes with the lowest P values across the C-NB/C-NBF and T-NBF/C-NBF comparisons for all *eYFP*^+^ neurons (M), *eYFP*^+^ mature neurons (N), and *eYFP*^+^ new neurons (O). Blue: genes with the same log_2_FC direction; orange: genes with opposite log_2_FC directions; dark blue/orange: T-NBF/C-NBF comparison; light blue/orange: C-NB/C-NBF comparison. **(P)** Percentage of same and opposite log_2_FC directions of genes shown in M–O. Significance: ***P < 0.001.

To gain an insight into the profile of new neurons versus mature neurons in the engram, we first examined their distribution based on their spatial transcription and observed reduced distribution of both immature and mature neurons in the C-NBF group compared to the C-NB and T-NBF (Fig. 10, A–C; and Fig. S4, A and B). We next examined the mean count of cells expressing the genes with the lowest P < 0.05 for each *eYFP*^+^ neuron type (Fig. 10, D–L). Among genes with P < 0.05, *Adam10*, *Mapk3*, *Gad1,2*, and *Slc17a8* were most modulated in the C-NB/C-NBF comparison for both *eYFP*^+^ neurons and *eYFP*^+^ mature neurons (Fig. 10, D and E). *Apoe*, *Bdnf*, *Camk2a*, *Nefh*, and *NeuroD1* were most modulated in *eYFP*^+^ immature neurons (Fig. 10 F). Interestingly, in the T-NBF/C-NBF condition, *Adam10* and *App*, *Camk2a*, *Glul*, and *Lmo1* were found to be most modulated in *eYFP*^+^ total neurons and mature neurons (Fig. 10, G and H), while *Apoe*, *Bdnf*, *Gabra1*, *Homer1*, and *Mapt* were the most modulated in new neurons (Fig. 10 I). In the T-NBF/C-NB condition, *Camk2a* and *Mapk3* were the most modulated in mature and immature neurons (Fig. 10, J–L). For log_2_FC of these genes, see Fig. S5, D–L. Comparing the directivity of the differentially expressed genes in the engram cells in C-NB/C-NBF and T-NBF/C-NBF revealed similar directionality (Fig. 10, M–O). Importantly, among the combined 20 genes with the lowest P values across the C-NB/C-NBF and T-NBF/C-NBF comparisons (Fig. 10, M–O), there was a statistically significant proportion that had the same fold change direction across the C-NB/C-NBF and T-NBF/C-NB comparisons than expected due to random chance alone for *eYFP*^+^ neurons (0.82, 95% CI = [0.645, 0.930]; P < 0.001), *eYFP*^+^ mature neurons (0.84, 95% CI [0.672, 0.947]; P < 0.001), and *eYFP*^+^ new neurons (0.93, 95% CI [0.786, 0.992]; P < 10^−6^; Fig. 10 P). Taken together, these results suggest that augmenting neurogenesis promotes a similar gene profile in the engram cells in FAD compared to the wild type (C-NB).

## Discussion

This study provides several novel observations. First is the direct evidence that immature neurons in the DG play a role in hippocampus-dependent memory engram in wild-type and FAD mice. Second, impairments in hippocampal neurogenesis cause defective engram formation in FAD and underlie memory deficits. Third, an increasing level of neurogenesis rescues memory by restoring the engram. Fourth, immature neurons are required for proper memory formation in FAD. Fifth, augmenting neurogenesis rescues deficits in spine density in both immature and mature engram neurons in the DG of FAD mice. Sixth, augmenting neurogenesis modulates the profile of immature and mature engram neurons in the DG to resemble the transcription profile of engram cells in wild-type mice. Seventh, AD-linked signals, particularly *App*, *Apoe*, and *Adam10*, play a role in the engram and are modulated following augmentation of neurogenesis and rescue of memory.

The direct connection of new neurons in the DG with the most vulnerable neurons in AD, namely, neurons in layer II of the entorhinal cortex, coupled with their high plasticity, led us to hypothesize that impairments in neurogenesis may compromise memory formation in AD. Adult-born granule cells regulate the relative synaptic strength of entorhinal neurons to shape distinct neural representations in the DG (Luna et al., 2019). Thus, it is reasonable to hypothesize that the fate of neurogenesis has a direct impact on hippocampus-dependent memory formation and its impairment in AD. Indeed, we show that immature neurons get recruited into spatial and contextual recognition memory circuits during the acquisition and retrieval of the memory in FAD mice. Immature neurons are functionally distinct from mature granule neurons in the DG. They are characterized by a lower threshold for the induction of long-term potentiation and a lower activation threshold (Kempermann et al., 2003; Schmidt-Hieber et al., 2004). In fact, by 4 wk of age, they are more likely than older granule cells to be recruited into circuits supporting spatial memory in wild-type mice (Gu et al., 2012; Kee et al., 2007). Our results support the notion that immature neurons are preferentially recruited into the memory circuit (Kee et al., 2007). In addition, we showed that immature neurons were favorably reactivated during memory retrieval. Thus, deficits in neurogenesis as observed in AD were significant for proper memory formation, leading to the recruitment of significantly fewer immature neurons into the engram. Our results are in agreement with this timeline and show that inactivating immature neurons at 4 wk of age disrupts memory. This strongly suggests that the additional immature neurons recruited into the engram in the T-NBF mice were required for a functional engram. Notably, spatial transcriptomics revealed that the total number of cells in the DG was significantly reduced in the C-NBF group compared to C-NB and T-NBF, but comparable in the C-NB versus T-NBF. This suggests that deficits in neurogenesis in FAD lead to reduced cell number in the DG, and augmenting neurogenesis restores the number of cells. This may have implications for DG function. In that regard, we use the term “immature neurons” to describe 4-wk-old newly maturing neurons and discriminate them from new neurons that are being continuously added to the granular layer of the DG throughout life.

It should be noted that in contrast to previous reports about a potential neurogenic toxicity of certain AAV serotypes, we have not observed any toxic effect of the viral engram kit cocktail used in this study. It is possible that we do not see the effect described in Johnston et al. because the serotype we used (AAV9) is different than the ones used in their study (AAV1,2,8). Different serotypes may have a distinct attachment receptor, capsid composition, and unique mechanism of endocytosis into the target cell. For example, AAV2 and AAV9 use different glycan moieties for initial attachment to the cell surface (Meyer and Chapman, 2022; Miyake et al., 2012; Pillay et al., 2017). Previous studies report that different serotypes exert different levels of toxicity on different cell types and that AAV9 particularly, exerts no toxicity on neurons and glia (Haggerty et al., 2020; Howard et al., 2008). However, we cannot exclude the possibility of an effect of the AAV on the neural stem cell population, which would not have been apparent in this study due to the 2-wk duration of the virus in vivo.

A previous study used an optogenetic approach to interrogate stage-specific memory formation deficits in FAD and found that memories were acquired in the FAD model, yet these were not retrieved under normal physiological conditions but only following optogenetic activation of the engram in the DG (Roy et al., 2016). However, this study did not discriminate between immature and mature neurons in the engram. Our study shows that immature neurons play an important role in the engram. While it is clear that immature neurons integrate in the hippocampal circuitry (Miller and Sahay, 2019), our study is the first to show that impairments in hippocampal neurogenesis play a role in memory formation in AD by depriving the availability of immature neurons for engram formation.

In addition to its effect on the number of new neurons recruited into the memory circuit, we show that the characteristics of immature neurons recruited into the engram are compromised in FAD and rescued following augmented neurogenesis. We show that the density of dendritic spines in FAD is reduced compared to wild-type mice and rescued following augmentation of neurogenesis. The density of dendritic spines and their structure are profoundly implicated in learning and memory, cognitive resilience, and cognitive deficits in neurodegenerative disorders (Chidambaram et al., 2019; Walker and Herskowitz, 2021). Interestingly, we also observed that the complexity of the dendritic tree of new neurons in the engram is compromised in FAD and that augmenting neurogenesis results in a larger and more developed dendritic tree of new neurons and mature granule neurons that incorporate into the memory circuit. Hierarchical branching pattern has long been associated with higher synaptic growth in DG neurons and plays an important role in pattern separation (Chavlis et al., 2017). In AD patients, granule cell dendrites have been found to be shorter and less branched with fewer spines in comparison to age-matched healthy controls (Reimann et al., 2017). Dendritic branching is a dynamic process regulating distance-dependent and high-order connectivity (Reimann et al., 2017). Thus, higher order branching is indicative of a greater likelihood of the neuron playing a role in the synaptic circuit (Reimann et al., 2017). This result is in agreement with our previous report that FAD-linked loss of PS1 function results in a compromised dendritic tree of new hippocampal neurons (Bonds et al., 2015). Future studies should address the manifestations of augmented neurogenesis on synaptic plasticity and the level of neuronal vulnerability of entorhinal cortex layer 2 (ECXII). A caveat in addressing this association is the lack of pronounced neuronal cell death in most mouse models of FAD. Notably, we observed that augmenting neurogenesis rescued deficits in spine density of mature granule neurons that play a role in the engram. This suggests that enhancing neurogenesis in FAD restores deficits in the DG, which may have major implications for the viability of ECXII neurons.

The association between the hallmarks of AD, namely, amyloid deposition and neurofibrillary tangles, and memory deficits remains controversial. We have not observed a change in the amount of amyloid deposition in T-NBF compared to C-NBF (data not shown). Several studies demonstrated a link between tau pathology and vulnerability of ECXII neurons (Fu et al., 2019; Fu et al., 2017). Particularly, the lateral entorhinal cortex (LEC) was identified as affected in preclinical stages and may induce the spread of pathology (Khan et al., 2014). Interestingly, a recent study observed the accumulation of phosphorylated tau in GABAergic interneurons of the DG of AD patients and mice, and that tau pathology impairs neurogenesis by downregulating GABA and disinhibiting excitatory circuitry neurons (Zheng et al., 2020). Consistently, we and others have shown that impaired neurogenesis through unknown mechanism, but suggestively due to reduced inhibitory tone in the DG resulting in its overexcitation (Hollands et al., 2017; Sun et al., 2009).

Our study shows that enhancing neurogenesis in the intact young adult brain does not further improve performance in the CFC or NOL tasks. This may have several explanations. First, a ceiling effect, where the addition of extra new neurons will not improve what is already an intact learning process. Second, the CFC and NOL are not cognitively demanding for the exquisite role of the extra newly mature neurons for the capturing of further observable improvement. Indeed, previous studies showed that enhanced neurogenesis significantly improves pattern separation (Sahay et al., 2011). Thus, at the mouse age tested here, augmentation of neurogenesis was effective in the case of deficiency of new neurons. In support of this notion, a recent study suggests that increasing neurogenesis compensates for aging-related reduced neurogenesis and hippocampus-dependent learning (Berdugo-Vega et al., 2020). Nevertheless, neurogenesis is severely impaired in AD patients and has mild cognitive impairments compared to normal aging (Tobin et al., 2019). Studies comparing the mechanism by which augmenting neurogenesis rescues learning and memory impairments in normal aging compared to AD are warranted. Our study provides novel insights into memory failure in AD and suggests that augmenting neurogenesis can rescue cognitive deficits in AD.

Beyond the activation of immediate early genes, the molecular profile of new neurons participating in memory circuits is not fully known. Moreover, the signaling pathways underlying defective neurogenesis in FAD and impaired recruitment of new neurons into the engram are yet to be fully determined. We and others have shown previously that cAMP response element binding protein phosphorylation (pCREB) and its complex is defective in FAD mice and AD patients (Bartolotti et al., 2016; Bartolotti et al., 2015). The activation of the CREB complex plays a major role in memory formation, as well as in the regulation of hippocampal neurogenesis (Beckervordersandforth et al., 2015; Jagasia et al., 2009; Ortega-Martinez, 2015). In addition, we have shown previously that PS1 regulates neural progenitor cell differentiation and that FAD-linked mutant PS1 compromises the function and morphology of new neurons incorporated in the granular cell layer of the DG (Bonds et al., 2015; Gadadhar et al., 2011). FAD-linked loss of function of PS1 leads to reduced levels of pCREB and phosphorylated β-catenin (Bonds et al., 2015). Similarly, we have demonstrated that APP plays a role in neurogenesis and regulates the survival and proliferation of neural progenitor cells and that FAD forms of APP compromise neurogenesis (Demars et al., 2013). Analyzing the profile of the engram revealed that the transcription of both new and mature neurons in the FAD differs from the wild type. Several genes stood out as the most significantly changed between the engrams of the different groups. Genes regulating cellular calcium, hippocampal and synaptic plasticity, such as *Camk2a*, *Mapk3*, *Pvalb*, and *Slc* family members, repeatedly exhibited inter-group differential expression. Genes regulating neurogenesis, synaptogenesis, and plasticity, such as *NeuroD1*, *Syt6*, *Ntn1*, *Tac2*, *Glul*, *Bdnf*, and *Rprm*, showed the largest differences. Of note, *App* was one of the most significantly changed between the engrams of T-NBF/C-NBF. Modulation of *App* expression was most apparent in mature neurons of the engram. Notably*,* *Adam10* was also one of the most significantly changing genes in the engram. *Adam10* is thought to have α-secretase activity and is implicated in both neurogenesis and AD (Demars et al., 2011; Demars et al., 2013; Lazarov and Demars, 2012; Toonen et al., 2016). *Adam10* and *App* show the most significant change following enhancement of neurogenesis in FAD mice. Interestingly, *Adam10* and *App* are of the most changed genes in mature engram neurons when comparing both C-NB/C-NBF and T-NBF/C-NBF, suggesting that enhancement of neurogenesis modulates similar pathways in the engram to those in the wild-type mice. While *App* has been implicated in neurogenesis and synaptic plasticity previously, this is the first demonstration of its role in the engram and memory formation. The robust change in the profile of mature neurons in the engram following augmentation of neurogenesis suggests that this intervention affects the hippocampal circuitry.

Most unexpected is the involvement of AD-related *Apoe* and *Adam10* genes in the engram. Intriguingly, *ApoE* was most significantly differentially expressed in new neurons in the engram. In new neurons, *ApoE* and *Bdnf* were the most changing between C-NB and C-NBF. While deficit in *Bdnf* has been implicated in impairments in neurogenesis in FAD, this is the first time that *ApoE* is implicated in neuronal function and memory formation in the engram. In addition, *Gabra1*, *Grin1*, and *Camk2a*, known to play a major role in neurogenesis and synaptic function (Fuchs et al., 2013; Goncalves et al., 2016; Samarut et al., 2018), were the most differentially expressed in new neurons in the engram. Future experiments will validate the functional role of these genes in DG neurons recruited to the engram. Most importantly, our results show that augmenting neurogenesis modulates the transcription profile of engram neurons in the DG in FAD to resemble one of the engram neurons in the DG of wild-type mice. This suggests that augmenting neurogenesis rescues memory deficits in FAD by affecting the molecular profile of the engram in the DG.

## Materials and methods

### Generation of mouse lines

All animal experiments were approved by the University of Illinois at Chicago Institutional Animal Care and Use Committee, ACC Protocol # 17–123 (Lazarov). Mice were housed in a 12-h (06:00–18:00) light–dark colony room with ad libitum food and water. *NestinCreER*^*T2*^ and *Bax* ^*fl/fl*^ transgenic animals were obtained from Dr. Rene Hen (Departments of Neuroscience and Psychiatry, Columbia University, New York, NY). 5XFAD mice were procured from The Jackson Laboratory (cat# 034848). *NestinCreER*^*T2*^ and *Bax*^*fl/fl*^ mice were bred to yield *Nestin; CreER*^*T2*^*; Bax*^*fl/fl*^ mice. *Nestin CreER*^*T2*^; *Bax*^*fl/fl*^ were bred with the 5XFAD mouse model to yield *NestinCreER*^*T2*^; *Bax*^*fl/fl*^; 5XFAD mice. All the animals were maintained on C57bl/6 genetic background. Experiments used female mice only. *Bax*^*fl/fl*^; 5XFAD mice generated from these crosses were used to assess CreER^T2^-independent effects of tamoxifen on behavior. Recombination was induced by intraperitoneal injection of 2 mg of tamoxifen (Sigma-Aldrich; T-5648–dissolved in 20 mg/ml concentration in corn oil), once a day for 5 consecutive days at the age of 4 wk. Control groups received a similar volume of corn oil intraperitoneally once a day for 5 consecutive days.

### Behavioral testing

Behavioral testing was performed 3.5 mo following tamoxifen or vehicle injection. All experiments and analyses were performed blindly to genotype or treatment.

#### NOL test

Mice were habituated in an empty 50 × 50 × 50 cm^3^ chamber for 5 min for 2 d. On day 3, two similar objects were placed at two corners of one side of the box, 10 cm apart. The animals were allowed to explore the chamber for a maximum of 20 min or until the animals explored the two objects for a total of 30 s. “Exploring the object” was defined as the head direction pointing toward the object, while the animal is touching the object. On day 4, one of the objects was placed in a new location diagonally to the other one. The animals were allowed in the chamber for up to 20 min or until the two objects were explored for 30 s. The video was captured for every run and analyzed by Ethovision 10 software for time spent with each object. The box was cleaned with 70% alcohol between trials. The discrimination index was calculated as *DI* = (*T*_N_ −*T*_O_)/(*T*_N_ + *T*_O_), where *DI* = discrimination index, *T*_N_ = time spent exploring the object at the new location, *T*_O_ = time spent exploring the old location.

#### CFC

This test was conducted in a 17.8 × 17.8 × 30.5 cm^3^ chamber encased by isolation cubicles. The context had two plexiglass walls, two metal walls, and a stainless-steel grid floor (Coulbourn Instruments). On day 1 of the test, animals were placed in the chamber for 180 s. A 2-s foot shock of 0.65 mA was administered at the 148th s. Mice remained in the arena for 30 s to associate the context with the shock and then placed back in their home cages. On day 2 the animals were placed in the same chamber for 5 min without shock treatment. The motion of animals was captured by a digital video camera mounted above the test cage. FreezeFrame and FreezeView software (Actimetrics) were used for recording and analyzing the freezing behavior of the animals. Previously, manual scoring sessions carried out by investigators blinded to conditions were shown to be consistent with Freeze Frame scoring system. Then 70% ethanol was used to clean the grid floor and walls between runs.

To test the effect of new neuron inactivation, 4 wk following stereotaxic injection of RV-HM4i-eGFP or RV-eGFP viral constructs into the DG, T-NBF mice were subjected to CFC, as above. To test for the overall effect of inhibiting new neuron activation on memory formation, mice received CNO 5 mg/kg (Cat # HB6149; Hellobio) in drinking water starting 5 d before training and throughout testing. The calculation for CNO dosage was made based on the weight and the amount of water an individual mouse consumed daily so that each mouse would ingest about 5 mg/kg concentration of CNO per day. The amount of freezing was assessed by FreezeFrame software by Coulbourn.

#### Light–dark test

Light–dark test was carried out to assess any anxiety-like behavior in animals. The light–dark test (*N =* 12–16 per group) was conducted in a box having a light chamber with clear walls and a dark chamber with one third of the total box size. The dark chamber is opaque to visible light. Both the chambers were connected with an opening at floor level in the center to allow passage between the light and dark compartments. The light compartment was brightly illuminated. The animals were kept in dark for at least 1 h before testing. The apparatus was cleaned between trials. The mice were kept in the light chamber to freely explore both chambers for 5 min. Time spent in each chamber was assessed.

### Engram viral cocktail

AAV9-cFos-tTA and AAV9-TRE-ChR2-eYFP were obtained from Dr. Susumu Tonegawa (Howard Hughes Medical Institute, Massachusetts Institute of Technology, Cambridge, MA) and were packaged as previously described (Roy et al., 2016). Viral packaging was carried out at the Vector core facility of the University of Massachusetts Medical School Gene Therapy Center. Viral titers were 1.5 × 10^12^ genome copy ml^−1^ for AAV9-c-fos-tTA and 1.2 × 10^12^ for AAV9-TRE-ChR2-eYFP were used in this study.

### Viral stereotaxic injection

#### Tagging engram neurons

Surgeries were performed at 4 mo of age and 2 wk before CFC. Mice were fed doxycycline-supplemented diet (40 mg/kg) starting 1 d before engram viral kit injection. Mice were anesthetized with 2.5% isoflurane and 100% oxygen mixture. Buprenorphine (0.01 mg/kg of body weight of animals) was given before the start of surgery. The two viruses were mixed in equal volume and 1 μl of this viral cocktail was injected bilaterally into the DG (anterior-posterior [AP] = −2.92 mm; medial-lateral [ML] = ±2.05 mm); dorsal-ventral [DV] = −2.5 mm) at a speed of 70 nl min^−1^ using 1 μl Hamilton microsyringe. The needle was lowered to the injection site and remained at the target coordinates for 5 min before injection. After the injection, the needle was kept for 10 min before being withdrawn to prevent the backflow of the virus cocktail. Animals were allowed to recover for 2 wk before behavioral experiments and kept on doxycycline-supplemented diet until the first day of CFC test to allow labeling of activated neurons during the memory acquisition phase. The animals were kept back on the doxycycline diet after the testing and were on diet for the remaining duration of the testing. All injection sites were verified histologically and immunohistochemically.

#### Manipulating the activation of new neurons using DREADD

Mice at 5 wk of age were injected with tamoxifen (130 mg/kg) intraperitoneally for 5 consecutive days. At 3.5 mo of age, mice were anesthetized with 2.5% isoflurane using the VetTech incorporation apparatus and underwent a surgical procedure as described above, except this time they were injected with retrovirus carrying either -pMMLV-CAG-EGFP-p2A-hM4, or RV-pCAG-EGFP (2.6 × 10^7^ titer, a gift from Jenny Hsieh, University of Texas at San Antonio, San Antonio, TX), or pMMLV-CAG-Tdtomato-P2A-hM4D(Gi; Vetor Builder) 2 μl into two sites of the dorsal DG with following coordinates, site 1: (AP = −1.8; ML = ±1.15, DV = −2.15), site 2: (AP = −2.55, ML = ±2.00, DV = −2.25) bilaterally. Mice were allowed 3 wk of recovery period. 3 wk after surgery, mice were gently handled twice a day for 7 d before the CFC experiment. The CFC experiment was carried out 4 wk after virus injection based on previous findings that this time is the most optimal time window to manipulate the functions of the newborn cells (Gu et al., 2012).

### BrdU injection

BrdU (B5002; Sigma-Aldrich) was dissolved in 0.9% NaCl at a concentration of 10 mg/ml and injected at 100 mg/kg of body weight three times a day for three consecutive days at the age of 3 mo and 1 wk. 5 wk later, the animals were perfused, brains were sectioned, and the number of BrdU^+^ cells and their identity were determined.

### Immunohistochemistry

Mice were deeply anesthetized by isoflurane and perfused transcardially with PBS followed by 4% paraformaldehyde. Brains were dissected and immersed in 4% paraformaldehyde overnight at 4°C followed by 30% sucrose solution for 3 d. Brains were sectioned (50 μm) coronally by a microtome (SM2010R; Leica) and stored −20° in a cryoprotectant solution. For immunostaining, sections were washed with 1× TBS three times, followed by blocking and permeabilization buffer (1× TBS + 0.25% Triton X-100 + 5% normal donkey serum) for 1 h. Sections were then incubated with primary antibodies at 4°C for 24 h. Following that, sections were washed with 1× TBS three times for 5 min each followed by 2 h of incubation with secondary antibodies. Sections were washed again in 1× TBS three times, 5 min each time, and counterstained with DAPI solution for 5 min. Sections were washed again twice for 5 min each, mounted on microscope slides, and topped with PVA-DABCO and coverslips. Images were taken on a confocal microscope (LSM 710; Zeiss). Analyzers were blind to the experimental conditions. Antibodies used for immunostaining were as follows: mouse anti-DCX (1:50, sc-271390; Santa Cruz), rabbit anti-DCX (1:250, Ab18723; Abcam), mouse anti-NeuN (1:400, MAB377; Millipore Sigma), rat anti-BrdU (1:250, Ab6326; Abcam), goat anti-GFP (1:500, Ab5450; Abcam), rabbit anti–c-fos (1:250, ab190289; Abcam), and rabbit anti–Egr-1 (1:250, A7266; Abclonal). For mouse DCX and rat BrdU antibodies, antigen retrieval with 10 mM sodium citrate solution was performed before blocking. All secondary antibodies were obtained from Jackson ImmunoResearch laboratory. For secondary antibodies, anti-mouse cy-3 and cy-5 (both 1:500), anti-rabbit cy-3 and cy5 (both 1:500), anti-goat Alexafluor-488 (1:1,000), and anti-rat cy-3 and cy-5 (both 1:500) were used.

### Confocal microscopy and stereology

Every sixth coronal section (50 μm) spanning through the whole DG region and 300 μm apart was used for cell count. Unbiased stereology was performed as previously described (Bonds et al., 2019). Briefly, cell counts were performed using design-based stereology (StereoInvestigator; MBF Biosciences). Brain sections were quantified using the optical fractionator workflow of StereoInvestigator. Regions of interest were traced under 5× magnification with counting performed under 63× magnification with a counting frame of 225 × 145 µm and sampling grid of 100 × 100 µm with 12.5 µm top and bottom guard zones. Alternatively, modified optical dissector method was used for unbiased quantification of the number of different cell types in DG (Semerci et al., 2017). Optical sections were scanned using confocal microscope (Zeiss LSM 710) in Z-stack at 25× objective. 3D reconstructions were obtained using the Zen 2.3 lite version.

### Dendritic spine analysis

Tertiary dendrites of triple-positive cells (DCX^+^eYFP^+^Egr1^+^) were sampled and quantified in 30 μm perfused and fixed brain sections of C-NB, C-NBF, and T-NBF mice using Neurolucida 360 software. The spine classification algorithm was used as previously described (Dickstein et al., 2016). Spines were classified based on several morphological criteria: head to neck ratio, length to head ratio, mushroom head size, and filopodium length.

### Statistical analysis

All statistical analyses were done in GraphPad Prism (Version 7.01; GraphPad Software Inc.) or Microsoft Excel. Statistical significance was assessed by Kruskal–Wallis test with Dunn’s post hoc test unless otherwise noted. One-variable experiments were analyzed by the two-tailed unpaired *t*-test. All data shown represent mean ± SEM and a probability of <0.05 was considered statistically significant.

### Tissue collection for in situ sequencing analysis

After 30 min of CFC testing, animals were deeply anesthetized with isoflurane followed by transcardial perfusion with ice-cold 1× PBS RNA-free (AM9625; Invitrogen) for 2 min. Brains were quickly isolated and placed gently into the sagittal brain matrix (# RBMS-200S; Kent Scientific) and trimmed sagittally. This approach allowed two brains to fit into a 1 cm^2^-size mold for cryosectioning (catalog # 62527-16; Electron Microscopy Sciences). Brains were applied with optimal cutting temperature compound (catalog # 23-730-571; Fisher) at the base for stability, while the base mold was on dry ice. The brain blocks were placed in a bigger peel-away and the optimal cutting temperature compound was applied until solidified as a block ready for cryosectioning. Fresh frozen brain blocks were placed in −80°C. Blocks were cryosectioned on a cryostat (CM3050; Leica) at 10 μm thickness and mounted on Superfrost Plus Microscope slide (# 12-550-15; Thermo Fisher Scientific). Brain sections were stored at −80°C until shipment. The quality of RNA extracted from brain sections was checked and confirmed that each sample had RNA integrity number >7 (Tapestation 4200; Research Resources Center, University of Illinois at Chicago). To validate the feasibility of quenching autofluorescence background, TrueBlack (23007; Biotium) was used for 30 s at room temperature. Brain sections were shipped to Cartana/10x Genomic (Sweden) for in situ sequencing.

### Library preparation, barcode sequencing, imaging and data processing

All samples, as 10 μm cryosections placed on SuperFrost Plus slides, were sent to CARTANA Sweden (part of 10x Genomics) for library preparation, in situ barcode sequencing, imaging. and data processing. Briefly, tissues were fixed for 5 min, permeabilized with 0.1 M HCl and quenched with TrueBlack for 30 s. For library preparation, chimeric padlock probes (targeting directly RNA and containing an anchor sequence as well as a gene-specific barcode) for three pre-defined panels (47, 31, 40 genes; see Genes of interest) as well as a custom panel of 41 genes were hybridized overnight at 37°C, then ligated before the rolling circle amplification was performed overnight at 30°C using the HS Library Preparation kit for CARTANA technology and following manufacturer’s instructions. All incubations were performed in SecureSeal chambers (Grace Biolabs). Optimal RNA integrity and assay conditions were also controlled using Malat1 and Rplp0 housekeeping genes by using the same protocol on serial sections. Quality control of the library preparation was also performed by applying anchor probes to detect simultaneously all rolling circle amplification products from all genes in all panels. Anchor probes were labeled probes with Cy5 fluorophore (excitation at 650 nm and emission at 670 nm). For barcode sequencing, adapter probes and sequencing pools (containing four different fluorescent labels: Alexa Fluor 488, Cy3, Cy5, and Alexa Fluor 750) were hybridized to the padlock probes to detect the gene-specific barcodes through a sequence-specific signal for each gene-specific rolling circle amplification product. This was followed by imaging and performed six times in a row to allow for the decoding of all genes in the panels. Raw data consisting of 20× images from five fluorescent channels (DAPI, Alexa Fluor 488, Cy3, Cy5, and Alexa Fluor 750) were each taken as the *z*-stack and flattened to 2D using maximum intensity projection. After image processing and decoding, the results were summarized in a csv file and gene plots were generated using MATLAB.

### Genes of interest

There were 159 genes of interest probed for in situ sequencing analysis: *Ache*, *Acta2*, *Adam10*, *Adamts2*, *Adarb2*, *Adora2a*, *Aldoc*, *Apoe*, *App*, *Aqp4*, *Arc*, *Arhgap36*, *Arntl*, *Atf4*, *Atxn1*, *Bace1*, *Bcl11b*, *Bdnf*, *Calb1*, *Calb2*, *Calca*, *Camk2a*, *Camk4*, *Cav1*, *Cblb*, *Cbln2*, *Cck*, *Ccr5*, *Cebpa*, *Chat*, *Chodl*, *Chrna2*, *Chrna6*, *Cnmd*, *Cpa6*, *Creb1*, *Crh*, *Crhr1*, *Crhr2*, *Crispld2*, *Cux1*, *Cux2*, *Dcn*, *Dcx*, *Deptor*, *Dicer1*, *Drd1*, *Egfr*, *Egr1*, *Eif2ak4*, *Eif2s1*, *Fev*, *Fezf2*, *Fos*, *Foxp2*, *Gabra1*, *Gabra2*, *Gad1*, *Gad2*, *Gfap*, *Gja1*, *Gls*, *Glul*, *Grin1*, *Grin2a*, *Grin2b*, *Hdac2*, *Homer1*, *Hpse*, *Igfbp4*, *Itgam*, *Kcnj8*, *Krt73*, *Lamp5*, *Laptm5*, *Lhx6*, *Lmo1*, *Lypd1*, *Map2*, *Mapk3*, *Mapt*, *Mbp*, *Mgll*, *Mmp9*, *Mup5*, *Ncam1*, *Ndnf*, *Nefh*, *Nes*, *Neurod1*, *Neurod6*, *Nf1*, *Npas4*, *Npy*, *Npy2r*, *Nrgn*, *Nrtn*, *Nt5c1a*, *Ntn1*, *Ntrk2*, *Nts*, *Oprk1*, *Oxt*, *P2rx3*, *Pcp4*, *Pdyn*, *Penk*, *Per1*, *Plat*, *Plch2*, *Plcxd2*, *Plp1*, *Ppp1r1b*, *Prox1*, *Psen1*, *Pthlh*, *Ptn*, *Ptprc*, *Pvalb*, *Rbbp4*, *Rbfox3*, *Reln*, *Rorb*, *Rprm, Rspo4*, *S1pr1*, *Satb2*, *Scn2a*, *Sema3e*, *Slc17a6*, *Slc17a7*, *Slc17a8*, *Slc6a1*, *Slc6a3*, *Slc6a4*, *Slc6a5*, *Sncg*, *Spp1*, *Sst*, *Syn1*, *Syt6*, *Sytl1*, *Tac1*, *Tac2, Tafa1*, *Tbr1*, *Th*, *Tpbg*, *Tph1*, *Trem2*, *Trh*, *Trhr*, *Trpv1*, *Tubb3*, *Unc5c*, *Vip*, *Vipr2*, *Wfs1*, and *eYFP*.

### Cell segmentation

To assign reads to individual cells, the DAPI nuclei images were cell segmented using a custom MATLAB script (Qian et al., 2020). Briefly, the nuclei images were divided into 2,000 × 2,000 pixel tiles, thresholded, and a watershed algorithm (Meyer, 1994) was used to detect cell boundaries and centroids assuming an ≈10 μm radius between cells. Reads within cell boundaries were then assigned to the cell centroid they were closest to. This was used to generate a gene by cell matrix for each sample, where each value is the number of reads for a given gene for each cell.

### DG masking

To study cells exclusively in the DG, we used the DAPI microscopy image for each section to manually outline the DG (including the hilus) using the polygonal selection tool in FIJI 1.53c (Schindelin et al., 2012). The (x, y) coordinates of the DG polygon masks were used to filter cells whose (x, y) coordinates were located within the DG mask. Quality control for viral injection was done by manual inspection of the spatial distribution of eYFP^+^ cells in each hemisphere and selecting those with the expected distribution in the DG for downstream analysis.

### Cell type classification

Cells were defined as “neurons” if they had zero reads for all of the following: *Acta2*, *Aldoc*, *Aqp4*, *Dcn*, *Gfap*, *Gja1*, *Itgam*, *Kcnj8*, *Laptm5*, *Mbp*, *Plp1*, *S1pr1*. Neurons were defined as “immature” if they contained at least one read for either *Dcx*, *Ncam1*, or *Neurod1*, or if they contained at least one read for *Prox1* if *Rbfox3* was not present. All other neurons were defined as “mature”. Cells were defined as *eYFP*^*+*^ if they contained at least one *eYFP* read.

For excitatory–inhibitory analyses, neurons within the DG granule cell layer were defined as “inhibitory” if they expressed at least one read for *Prox1*, *Rbfox3*, *Map2*, *Syn1*, or *Tubb3*, and expressed at least one read for any of the inhibitory gene markers *Adarb2*, *Arhgap36*, *Calb1*, *Calb2*, *Cck*, *Chodl*, *Chrna2*, *Cnmd*, *Crh*, *Crhr2*, *Crispld2*, *Gabra1*, *Gabra2*, *Gad1*, *Gad2*, *Hpse*, *Igfbp4*, *Krt73*, *Lamp5*, *Lhx6*, *Lmo1*, *Npy*, *Nrtn*, *Nts*, *Plch2*, *Pthlh*, *Pvalb*, *Rspo4*, *Sema3e*, *Sncg*, *Sst*, *Tac1*, *Tac2*, *Tafa1*, *Tpbg*, *Vip*, or *Vipr2*. After defining the inhibitory neurons, the remaining population of mature neurons was defined as “excitatory.”

### Differential expression analysis

FET was used to compare the proportion of cells expressing each gene across pairwise groups for each cell type. Contingency tables were constructed for each gene by first pooling cell counts from each sample within the group and calculating the frequency of cells expressing/not expressing a given gene for each group. The percent of cells expressing each gene in each group was used to calculate the log_2_ fold change (log_2_FC). To account for cases with zero gene expression and avoid ± infinity log_2_FC values, the smallest non-zero percent expression value across all genes was added to both groups as a pseudovalue. Benjamini-Hochberg false discovery rate was used to correct for multiple comparisons. Unless otherwise stated, unadjusted P values are shown.

### Fold-change consistency analysis

The union of the 20 genes with the lowest P values from FET for the C-NB/C-NBF and T-NBF/C-NBF comparisons were selected. For each gene, the log_2_FC was calculated as described above. The binomial test was used to determine if the probability of the observed proportion of log_2_FC with the same and opposite directions was statistically significant.

### Dissimilarity analyses (ANOSIM)

The fraction of cells expressing each gene was computed for each cell type (*eYFP*^*+/−*^, neuronal, and new or mature neurons) within each DG hemisphere. Euclidean distances were calculated using the vegan library (Oksanen et al., 2018) and tested for significance using ANSOIM. PCoA plots were generated in R using the ggplot2 library (Wickham, 2009).

### Software

MATLAB 9.0.0.341360 (R2016a) was used to perform cell segmentation. R version 3.6.3 (2020-02-29) was used for differential expression analysis and visualization.

### Online supplemental material

Supplementary information provides further validation measures of experimental models (Figs. S1 and S2), the foundation for dendritic spine analysis (Fig. S3), additional support information for the in situ sequencing analysis (Figs. S4 and S5), and a list of antibodies used in this study (Table S1).

## Supplementary Material

Table S1 lists antibodies used in this study.
