# Supplementary material for: Augmenting neurogenesis rescues memory impairments in Alzheimer’s disease by restoring the memory-storing neurons
Source: J Exp Med. 2022 Aug 19;219(9):e20220391. doi: 10.1084/jem.20220391 (PMC9399756; doi:10.1084/jem.20220391)
Supplement: Table S1 — lists antibodies used in this study. [file JEM_20220391_TableS1.docx]

**Table S1. Antibodies used**

| **Primary antibodies** | **Company** | **Catalog number** | **Dilution** |
| --- | --- | --- | --- |
| mouse DCX | Santacruz | sc-271390 | 1:50 |
| rabbit DCX | Abcam | Ab18723 | 1:250 |
| mouse NeuN | Millipore Sigma | MAB377 | 1:400 |
| rat BrdU | Abcam | Ab6326 | 1:250 |
| goat GFP | Abcam | Ab5450 | 1:500 |
| rabbit c-fos | Abcam | ab190289 | 1:250 |
| rabbit Egr-1 | Abclonal | A7266 | 1:250 |
| Rat GFAP | Thermo Fisher Scientific | 13-0300 | 1:1,000 |
| Rabbit Iba-1 | Fisher Scientific | 019019741 | 1:1,000 |
| **Secondary antibodies** | **Company** | **Catalog number** | **Dilution** |
| Donkey anti-mouse Cy3 | Jackson Laboratory | 715-165-151 | 1:500 |
| Donkey anti-mouse Cy5 | Jackson Laboratory | 715-175-151 | 1:500 |
| Donkey anti-rabbit Cy3 | Jackson Laboratory | 711-165-152 | 1:500 |
| Donkey anti-rabbit Cy5 | Jackson Laboratory | 711-175-152 | 1:500 |
| Donkey anti-goat Alexafluor-488 | Jackson Laboratory | 705-545-147 | 1:1,000 |
| Donkey anti-rat Cy3 | Jackson Laboratory | 712-165-153 | 1:500 |
| Donkey anti-rat Cy5 | Jackson Laboratory | 712-175-150 | 1:00 |
